# Course of neuropsychological health in post-COVID patients differs 6 and 12 months after inpatient rehabilitation

**DOI:** 10.3389/fpsyt.2025.1460097

**Published:** 2025-04-25

**Authors:** Katrin Müller, Iris Poppele, Marcel Ottiger, Rainer-Christian Weber, Michael Stegbauer, Torsten Schlesinger

**Affiliations:** ^1^ Department of Social Science of Physical Activity and Health, Institute of Human Movement Science and Health, Faculty of Behavioural and Social Sciences, Chemnitz University of Technology, Chemnitz, Germany; ^2^ BG Hospital for Occupational Disease Bad Reichenhall, Bad Reichenhall, Germany

**Keywords:** work-related COVID-19, post-COVID, mental health, depression, cognitive health, rehabilitation, long-term outcomes

## Abstract

**Background:**

Rehabilitation is an effective and feasible approach for post-COVID patients to improve mental health and cognitive complaints. However, knowledge regarding the long-term impact of rehabilitation on neuropsychological health of these patients is lacking.

**Objective:**

This study aims to investigate psychological health, fatigue, and cognitive function 6 and 12 months after inpatient post-COVID rehabilitation of patients, who acquired COVID-19 in the workplace. In addition, group differences in these outcome parameters according to sex, age, acute COVID status, socioeconomic status, profession, and pre-existing diseases will be detected.

**Methods:**

This longitudinal observational study examined the changes in mental and cognitive health of 127 patients with COVID-19 as an occupational disease or work accident. Symptoms of depression and anxiety, fatigue severity, somatic symptom severity, trauma-related symptoms, and cognitive functioning were assessed at the beginning as well as six and 12 months after rehabilitation. Group differences concerning sex, age, acute COVID status, socioeconomic status, occupational status, and existing diseases prior to COVID-19 were also analyzed.

**Results:**

The results showed that the improvements direct after rehabilitation in mental health and fatigue severity could not be maintained six and 12 months after rehabilitation discharge. Contrary, patients’ cognitive function maintained stable during follow-up. Significant group differences were observed regarding age, sex, acute COVID status, socioeconomic status, occupational status, and pre-existing diseases.

**Conclusion:**

This study highlights the importance of the aftercare process and the implementation of adequate and individualized therapeutic interventions such as psychological support and strengthen self-management skills.

The study is registered in the German Clinical Trials Register with the identifier DRKS00022928.

## 1 Introduction

An infection with SARS-CoV-2 is primarily known as respiratory disease but it can also impact other organs and develop into a multisystem disease. The course of disease is quite unspecific, has a broad symptom range, and can impact patients’ health and quality of life in a long term (1). According to national and international guidelines persistent manifestations and symptoms following an acute SARS-CoV-2 infection that cannot be justified by an alternative diagnosis are classified either into long-COVID (four weeks or longer) or post-COVID (more than 12 weeks) (2, 3). The known transmission ways of SARS-CoV-2 lead to increased risk of infection particularly in workplaces with intensive interpersonal and physical contacts with colleagues, patients and clients (4, 5). Especially healthcare-workers and social professionals showed a higher prevalence of SARS-CoV-2 compared to the general population (6), thus, it is necessary to highlight this vulnerable group in post-COVID research. In Germany, 357.396 cases of COVID-19 have been recognized as occupational diseases. Furthermore, 26.958 recognized cases of COVID-19 have been recorded as work-related accidents (according to the German Social Accident Insurance) (7).

### 1.1 Post-COVID and psychological symptoms

It is known that post-COVID can manifest in psychological symptoms, which can persist several months after acute SARS-CoV-2 infection (8–10). A systematic review from Marchi et al. (8) conclude, that 27-30% of patients experience sleep disturbances, 16-31% of patients experience symptoms of anxiety, 21-25% symptoms of depression, and 19-22% symptoms of PTSD. These prevalences are in line with the results of a recent systematic review and meta-analysis of Seighali et al. (9) including over 165 studies with 9.923.461 total post-COVID patients, reporting a pooled prevalence of depression and anxiety of 23% and of sleep disorders of 45%. A retrospective cohort study could reveal that the risk of experiencing mental health concerns (major depression, anxiety) is twice as high for COVID-19/Long-COVID survivors than for patients without history of COVID-19 (11). Overall, the authors of the above-mentioned studies also emphasize the high heterogeneity among existing studies in the prevalence of psychiatric symptoms e.g., due to heterogeneous study designs. Hyassat et al. (12) assessed post-COVID symptoms in a population of healthcare-workers with post-COVID and 58.4% of patients reported mood changes due to post-COVID. According to D’Ávila K et al. (13), Fouad et al. (14) and Mendola et al. (15) 8-44% of healthcare-workers are still showing symptoms of depression and anxiety after three to ten months of acute SARS-CoV-2-infection. Longitudinal studies highlight the long mental burden of COVID-19 and post-COVID. Fernandez-de-Las-Penas et al. (16) assessed symptoms of anxiety and depression with the hospital anxiety and depression scale (HADS) five (T1) and 10 (T2) months after hospital discharge in 2000 patients. The scores revealed that the prevalence of symptoms of anxiety decreased from 16.0% to 15.1% from T1 to T2, and symptoms of depression from 18.0% to 13.2%. Until know, there are some studies with follow-up times ≥ 24 months (17–19). In all studies the prevalence of anxiety and depression is decreasing during follow-up. With a prevalence of anxiety within 16.6-38.0% and of depression within 21.8-40.0% after 24 months after COVID-19. These findings emphasize that most patients are experiencing a long-lasting burden of disease with a recovery time of several months or years and long-term therapeutic concepts are needed.

### 1.2 Post-COVID and fatigue

Post-COVID patients are often reporting symptoms of fatigue (18, 20–22). According to a prospective registry study including 1.022 participants, around 30% of post-COVID patients fulfilled the criteria for ME/CFS after 255 days post infection (21). This prevalence is decreasing to 19.4% after 402 days post-infection. The assessed BFI (Brief Fatigue Inventory) score has a mean value of 5.4 points at visit 1 indicating moderate symptoms of fatigue. According to self-reported symptoms, 83% of patients have symptoms of fatigue. At visit 2 (402 days post infection) the mean BFI value is 5.0 and 73% of patients are reporting symptoms of fatigue. Van Wambeke et al. (18) report higher prevalence of fatigue (90%) 24 months after infection. However, the study sample was quite small with only 45 post-COVID patients. Another study with a median follow-up duration of 26 months since infection did reveal a prevalence of 21% for clinically relevant fatigue assessed by the FACIT Fatigue scale (23). There are only few studies assessing symptoms of fatigue in healthcare-workers. Hyassat et al. (12) assessed fatigue by self-reports as wells as the Fatigue Assessment Scale (FAS). After three months of SARS-CoV-2 infection 18.2% of healthcare-workers are reporting symptoms of fatigue which is decreasing to 10% after 6 months of infection. This prevalence is substantial lower than in the above-mentioned studies which presumably results from the relatively small and young study sample. According to the FAS results, female healthcare-workers are showing higher levels in fatigue than male healthcare-workers. In sum, the reported studies so far illustrate the high prevalences of fatigue which maintain stable over a long period of time. This should be addressed through adequate and patient-centered therapies.

### 1.3 Post-COVID and cognitive disturbances

Cognitive dysfunction is also one of the most reported symptoms of post-COVID patients (10, 19, 24). The prevalence of cognitive deficits in post-COVID patients is between 4-26% (8). A longitudinal study of Martin et al. (24) compared cognitive performance of post-COVID patients with matched healthy controls. Post-COVID patients showed cognitive slowing compared to control group. Further, there was no improvement in cognitive function after 6 months follow-up. Fernandez-de-Las-Penas et al. (25) examined cognitive function in hospitalized COVID-19 patients over 18 months and predicted recovery curves. While the prevalence of concentration loss and cognitive blurring is decreasing and will probably fall below 1% after two years, the prevalence of memory loss will remain high with over 10% after two years. A prospective study revealed a prevalence of cognitive impairments of 19% among post-COVID patients after 26 months post infection. The authors assume that the prevalence may be even higher as the used assessment tool (Montreal Cognitive Assessment (MoCA)) is not as sensitive as a more detailed cognitive test battery (23). There is also a high prevalence of cognitive complaints within healthcare-workers. After nine months of infection, 70.7% of healthcare-workers were reporting difficulties in concentrating and memory (26). Omar et al. (27) also revealed lower scores in Addenbrooke’s Cognitive Examination III for attention and memory in healthcare-workers with post-COVID compared to healthy healthcare-workers (control group). These findings underscore the importance of recognizing and addressing cognitive deficits in post-COVID patients to support their long-term recovery and health related quality of life.

### 1.4 Post-COVID and rehabilitation

To date, it is known that rehabilitation is a promising approach to improve post-COVID patients’ mental and cognitive health as well as quality of life (28, 29). A prospective study of Hayden et al. (30) observed improvements in symptoms of depression, anxiety and fatigue direct after rehabilitation discharge. Observations by Rutsch et al. (31) and Kupferschmitt et al. (32) confirm these results. The investigation of Müller et al. (33) revealed significant improvements in neuropsychological health and fatigue in patients who acquired COVID-19 in the workplace after an inpatient rehabilitation. In addition, healthcare-workers showed significantly greater improvements in depressive symptoms than non-healthcare-workers (33). Systematic reviews have also shown a positive effect of rehabilitation on psychological health (34–36). Three to six months after rehabilitation the scores for depression and fatigue decreased slightly but stayed on a higher level than before rehabilitation (30). The scores for anxiety decreased to baseline level (30). According to Gloeckl et al. (37) it is important to differentiate post-COVID patients after their predominant symptom to choose the most appropriate therapy and rehabilitation program. Several guidelines emphasize the importance of an interdisciplinary approach for the treatment and rehabilitation of post-COVID patients, especially in patients with psychological and cognitive ailments (2, 38–40).

To sum up, recent research confirms impaired mental health, symptoms of fatigue and cognitive deficits in post-COVID patients over two years after primary infection. This also applies healthcare-workers, which have a higher risk for getting infected with SRARS-CoV-2. As it is known that rehabilitation is a feasible and comprehensive therapy to improve patients’ mental health and cognitive impairments, there is limited knowledge regarding the long-term impact of rehabilitation on post-COVID in consideration of work-related infections, for example, in healthcare-workers. Longitudinal research data is needed to gain more knowledge about the long-term course of disease and to derive profound insights for an adequate after-care process of post-COVID patients. Thus, the current study investigates psychological health, fatigue, and cognitive function 6 and 12 months after inpatient post-COVID rehabilitation of patients, who acquired COVID-19 in the workplace. In addition, the study aims to detect group differences in these outcome parameters according to sex, age, acute COVID status, socioeconomic status (SES), profession, and pre-existing diseases.

## 2 Materials and methods

This research was undertaken at Chemnitz University of Technology, Germany, in collaboration with BG Hospital Bad Reichenhall. It was registered in the German Clinical Trials Register with the identifier DRKS00022928. Approval for the study was granted by the Ethics Committee of the Bavarian State Medical Association (number 21092) and the Ethics Committee of Chemnitz University of Technology (TU Chemnitz, Chemnitz, Germany), Faculty of Behavioural and Social Sciences (number V-427-17-KM-COVID-19-18022021). In the following sections, only pertinent information related to the present research question is presented. A comprehensive overview of the study and the inpatient rehabilitation program is given in the previously published study protocol (41).

### 2.1 Study design and participants

The longitudinal study design consists of four distinct measurement points at home as well as during the clinical stay: the beginning (T1) and end (T2) of the inpatient rehabilitation period, as well as 6 months (T3) and 12 months (T4) after the end of rehabilitation. Patients were recruited by a study nurse at the BG Hospital Bad Reichenhall after being registered for rehabilitation by the respective accident insurance provider. A majority of the post-COVID patients treated at this facility are employed in the healthcare sector, although other professional groups are also represented. Patients in the post-acute phase of COVID-19, recognized as an occupational disease or work-related accident, were eligible for inclusion if they met specific criteria (being in the post-acute phase without evidence of infectivity, confirmed capability for rehabilitation, and voluntary participation in the study), and provided written informed consent. The current report presents results of the measurement points T3 and T4. Results from the analysis of the measurement point T1 compared to T2 are already published elsewhere (33).

All participants went through an inpatient multidisciplinary post-COVID rehabilitation program at the BG Hospital with a mean duration of M=28.99 (± 5.22) days. In addition to medical treatment and care, patients received in comprehensive physical and psychological treatments by specialists. For detailed information on the inpatient rehabilitation, see Müller et al. (41).

The study included 127 patients at T1. At time point T2, three participants were classified as dropouts, either due to discontinuation of rehabilitation (n=1) or lack of further interest in study participation (n=2). At time point T3, three participants discontinued the study due to lack of interest. At time point T4, two participants discontinued the study without providing reasons, one participant lost interest in participation, one participant experienced a stroke shortly before the T4 assessment, one participant couldn’t complete the questionnaires or travel to the clinic in time, and one participant cited professional reasons for discontinuing participation at T4. Consequently, there are 121 patients in the paired sample T1-T3 and 115 patients in the paired sample for T1-T4. Due to missing values (reasons: e.g., missing questionnaire response) or missing clinical examination, cases for each variable vary between 107-118 for T1-T3 comparison and 98-114 for T1-T4 comparison.

At this point it should be mentioned, that after inpatient rehabilitation at BG Hospital Bad Reichenhall up to the time point T4 participants received the following post-COVID-19 treatments: 79% further medical treatment by the general practitioner, 23% repeated rehabilitation, 45% outpatient psychological therapy, 79% ambulatory physiotherapy, 15% exercise in an outpatient group (see **Table 1**).

**Table 1:** Sociodemographic data at T1.

	N = 121^1^	Mean	SD	NA^2^
Sex				-
Male	29 (24%)			
Female	92 (76%)			
Age [years]	-	50.62	10.87	-
BMI [kg/m²]				-
Normal	17 (14%)			
Overweight	39 (32%)			
Obesity class I	35 (29%)			
Obesity class II	20 (17%)			
Obesity class III	10 (8%)			
Smoking status				-
Currently (every day)	5 (4%)			
Currently (occasional)	4 (3%)			
Former	46 (38%)			
Never	66 (55%)			
COVID-19 severity				-
Mild/ Moderate	85 (70%)			
Severe	30 (25%)			
Critical	6 (5%)			-
Pneumonia due to COVID-19	36 (30%)			1
Interval COVID-19 to Rehabilitation [days]		409.52	143.97	-
Rehabilitation duration [days]	-	28.99	5.22	-
PCFS-Score before Rehabilitation				-
No limitations	11 (9%)			
Negligible limitations	2 (2%)			
Slight limitations	43 (36%)			
Moderate limitations	63 (52%)			
Severe limitations	2 (2%)			
Occupation				-
Healthcare-worker	85 (70%)			
Non-healthcare-worker	36 (30%)			
Socio-economic status				1
medium	41 (34%)			
high	79 (66%)			
Pre-existing diseases				-
Metabolic disease	79 (65%)			
Cardiovascular disease	58 (48%)			
Respiratory disease	52 (43%)			
Psychological disease	23 (19%)			
Neuro-sensory disease	40 (33%)			
Aftercare interventions				
Further medical treatment	96 (79%)			9
Repeated rehabilitation	28 (23%)			10
Exercising in an outpatient group	18 (15%)			16
Ambulatory physiotherapy	95 (79%)			11
Outpatient psychological therapy	54 (45%)			12

^1^n (%); ^2^NA, not available/ no answer.

The patients are m=50.62 years (± 10.87) old and n=97 (76%) categorize themselves as female. At T1, 85% are overweight (BMI >25 kg/m^2^). Six patients are smokers, four patients are occasional smokers, and 49 patients are former smokers. 67 patients did never smoke. Further, 85 patients are working within the healthcare-sector and 36 patients are classified as non-healthcare-workers (e.g., administrative staff, industrial-/building technicians, social education staff, and teachers). According to the SES, 41 patients are reaching a medium SES, and 79 patients are reaching a high SES. None of the patients were in the lowest SES category.

### 2.2 Measurements

Several socioeconomic and -demographic variables (e.g., age, sex, socio-economic status, education) were obtained via questionnaire based on the German Health Interview and Examination Survey for Adults (DEGS) (42, 43). Pre-existing diseases were assessed using the subscale “Current diseases” of the work ability index (WAI) (44), and were complemented by a semi-standardized interview by a physician during medical anamnesis. Additionally, the Post-COVID-19 Functional Status (PCFS) scale with ranges from 0 (no limitations/symptoms in everyday life) to 4 (severe limitations/symptoms in everyday life was also used (45).

The German version of the Hospital Anxiety and Depression Scale (HADS-D) was used to assess the presence of psychological symptoms. The subscales for depression (HADS-D_Depression_) and anxiety (HADS-D_Anxiety_) consist of seven items, respectively. A sum score for each scale was generated (range 0–21) based on a Likert scale from 0 (‘no symptoms’) to 3 (‘severe symptoms’). Scores between 8 and 10 indicated mild symptoms, while scores above 10 indicated moderate to severe symptoms (46, 47). Based on analyses with cardiac patients after rehabilitation, a minimal clinically important difference (MCID) of >1.7 points was reported in each subscale (48). The subjectively perceived status of mental health was reported by participants on a self-generated questionnaire with 10 items on a scale of 0–10 (0 = very bad, 10 = very well).

To measure various physical symptoms and their symptom severity in patients the self-administered Somatic Symptom Disorder - B Criteria Scale (SSD-12 (49)) was used. SSD-12 consists of 12 items that assess the extent and impact of somatic symptoms, and each item is rated on a Likert scale from 0 (never) to 4 (very often), resulting in a total score ranging from 0 to 48. Higher scores indicate greater severity and impact of somatic symptoms and the MCID is defined as a change of 3 points (50).

The International Trauma Questionnaire (ITQ (51)) is a self-reported measure to assess the severity, frequency, and duration of trauma-related symptoms in individuals. The ITQ consists of 6 Items, rated on a Likert scale from 0 (not at all) to 4 (extremely). The calculated total score ranges from 0 to 24. A cut-off score of 19 or above may indicate the presence of PTBS, while higher scores indicate a more severe level of trauma-related symptoms. There is no well-defined MCID for the ITQ in the current literature.

The Brief Fatigue Inventory (BFI) and the Fatigue Impact Scale (FIS) were used to assess symptoms of fatigue. The BFI consists of ten questions concerning fatigue and fatigue-related symptoms to detect fatigue severity. The mean score of all items ranges from 0–10, a higher score indicates a more severe level of fatigue. In addition, depending on the mean score, the following categories can be used: 0 for ‘no fatigue’, 1-3 for `mild fatigue`, 4-6 for `moderate fatigue` and 7-10 for `severe fatigue` (52, 53). The MCID for in BFI score is defined as a change of 1.33 points (54). The FIS includes 40 items with a five-point Likert scale from 0 (‘no problem’) to 4 (‘extreme problem’) assigned to the three subscales (cognitive, physical, and psychosocial functioning). In addition, a sum score is calculated (range 0-160), with higher scores indicating more severe function impairments due to fatigue (55). A study in patients with multiple sclerosis defined a MCID of 10-20 points in FIS sum score (56).

We used three German versions of the MoCA (T1: Version 8.1, T2: version 8.2, T3: version 8.3, T4: version 8.1) to assess global cognitive functioning, e.g., short-term memory, visuospatial abilities, attention, concentration, working memories, language, orientation in space and time, and executive functions. The total sum score, ranging from 0 to 30 points, was calculated in addition to classifying participants into cognitively healthy (26-30 points) and mildly cognitively impaired (20-25 points) individuals (57). The MCID for the MoCA is defined as a change of two points in the total sum score, which was used to assess significant improvements or decline in cognitive abilities (58). Furthermore, the Digit Symbol Substitution Test (DSST) was used to examine various cognitive functions, including sustained attention, visual–spatial skills, response speed, and set-shifting. Participants were given a sheet of paper with rows of symbols and the task of matching each symbol to a number. A legend at the top of the page indicated which symbol matches which digit (1–9). Afterward, the number of correct digit-symbol matches executed in 90 s was recorded (59). There is no well defined MCID of the change in DSST score in a clinical sample. However, Jehu et al. (60) defined a MCID of 3-5 symbols in a population of older adults. The Trail Making Test (TMT) is a neuropsychological assessment tool that assess the individual`s focus, attention, execution function, and cognitive flexibility. There are two parts to the TMT (1): TMT-A, participants have to connect numbers in an ascending order (2), TMT-B, participants have to connect numbers and letters in an alternating way (61). The MCID of TMT-A is 11.7 seconds and 24.4 seconds in cognitive unimpaired individuals (62).

### 2.3 Data analysis

The data were analyzed using SPSS software (version 29, SPSS Inc., Armonk, NY, USA). Given the non-normal distribution of most parameters, the Wilcoxon signed-rank test was used to compare variables across T1, T3, and T4. Group differences at T1 and over time (Δ=T3-T1, Δ=T4-T1) concerning sex (male vs. female), age (≤50 years vs. >50 years), profession (healthcare services vs. other), acute COVID status (mild/moderate vs. severe/critical), socio-economic status (medium SES vs. high SES), and diseases prior to COVID-19 (cardiovascular disease, respiratory disease, neuro-sensory disease, metabolic disease, mental impairment) were analyzed using the Mann-Whitney U test. Only significant results regarding group differences at each measurement time point are presented in the text. Missing data were noted and are clearly presented in the tables, and p-values <0.05 considered statistically significant. Effect sizes were reported as r, with an effect size of 0.1 representing a ‘small’ effect, 0.3 a ‘medium’ effect, and 0.5 a ‘large’ effect, following the guidelines of Fritz et al. (63).

## 3 Results

### 3.1 6 months after rehabilitation

At T1, 23.7% of patients had mild symptoms of depression (HADS-D_Depression_) and 27.2% showed symptoms of severe depression whereas at T3 17.5% of patients had mild symptoms of depression and 25.5% showed symptoms of severe depression (**Supplementary Figure 1**). For anxiety (HADS-D_Anxiety_), 19.3% had mild symptoms and 25.4% had severe symptoms at T1. In comparison, at T3 mild symptoms were found in 14.9% of patients and 18.4% had severe symptoms (**Supplementary Figure 2**). At T1 mild cognitive impairment (MoCA) was found in 26.9% of patients and at T3 in 31.5% (**Supplementary Figure 3**). At baseline, 61.0% of patients showed moderate and 23.7% severe symptoms of fatigue according to the BFI score. At T3, the prevalence was almost the same with 61.9% of patients with moderate and 23.7% of patients with severe symptoms in fatigue (**Supplementary Figure 4**).

**Table 2** presents the differences in the mental and cognitive health assessments between T1 and T3. From T1 to T3, a significant improvement in HADS-D_Anxiety_ was recorded (T1: Mdn=6.50, IQR: 4.00-11.00; T3: Mdn=6.00; IQR: 3.00-9.25; p=0.041) with a low effect (r=-0.191). ITQ also showed a significant change between T1 (Mdn=7.00, IQR: 3.00-14.00) and T3 (Mdn=6.00, p=0.006, r=-0.253). A significant change in SSD-12, depression (HADS-D_Depression_), fatigue (BFI, FIS), and subjective mental health was not recorded between the measurement points T1 and T3 (p>0.05). The patients’ cognitive performance did improve, although not significantly, from T1 to T3 measured by MoCA. However, executive functions in the context of working memory and processing speed improved (DSST1: T1: Mdn=46.00, IQR: 37.00-53.00; T3: Mdn=50.00, IQR: 44.00-57.00; p<0.001), as well as recall performance (DSST2: T1: Mdn=5.00, IQR: 3.00-6.00; T3: Mdn=6.00, IQR: 4.00-8.00; p<0.001) significantly with moderate effect size (r=0.454). TMT-A showed a significant change from T1 (Mdn=34.35, IQR: 27.00-43.68) to T3 (Mdn=30.75, IQR: 24.65-38.51; p=0.004, r=-0.280), as did TMT-B from T1 (Mdn=71.58, IQR: 59.33-89.22) to T3 (Mdn=69.55, IQR: 52.16-82.85; p=0.005, r=-0.268), both with low effect sizes. However, no significant change was observed in the ratio of TMT B/A (p>0.05).

**Table 2:** Differences in neuropsychological health and fatigue between T1 and T3.

	N	T1			T3			z	p	r
		Min	Max	Median (IQR)	Min	Max	Median (IQR)			
HADS-D_Depression_	114	0.00	19.00	8.00 (4.00 – 11.00)	0.00	21.00	6.50 (3.00 – 11.00)	-1.378	0.168	-0.129
HADS-D_Anxiety_	114	0.00	18.00	6.50 (4.00 – 11.00)	0.00	20.00	6.00 (3.00 – 9.25)	-2.041	0.041	-0.191
SSD-12	118	1.00	47.00	25.00 (17.00 – 32.00)	0.00	45.00	23.50 (17.75 – 33.00)	-0.683	0.495	-0.063
ITQ	117	0.00	22.00	7.00 (3.00 – 14.00)	0.00	22.00	6.00 (1.00 – 10.50)	-2.733	0.006	-0.253
BFI	118	0.67	8.89	5.55 (4.30 – 6.66)	0.00	9.11	5.88 (4.88 – 6.80)	1.329	0.184	0.122
FIS	117	0.00	155.00	96.00 (72.50 – 113.50)	0.00	154.00	94.00 (68.50 – 113.00)	-0,053	0,958	-0.005
MoCA	108	18.00	30.00	27.00 (25.00 – 28.00)	18.00	31.00	27.00 (25.00 – 28.00)	-1.146	0.252	-0.110
DSST1	107	11.00	84.00	46.00 (37.00 – 53.00)	13.00	86.00	50.00 (44.00 – 57.00)	4.703	<0.001	0.455
DSST2	107	0.00	9.00	5.00 (3.00 – 6.00)	0.00	9.00	6.00 (4.00 – 8.00)	4.703	<0.001	0.455
TMT-A	108	13.80	130.00	34.35 (27.00 – 43.68)	15.00	164.00	30.75 (24.65 – 38.51)	-2,911	0,004	-0.280
TMT-B	108	32.80	300.00	71.58 (59.33 – 89.22)	30.00	214.00	69.55 (52.16 – 82.85)	-2.782	0.005	-0.268
Subj. mental health	118	0.82	9.36	5.27 (4.45-6.36)	1.73	9.55	5.09 (4.27 – 6.63)	-0.170	0.865	-0.016

HADS-D, German Hospital Anxiety and Depression Scale; SSD-12, Somatic Symptom Disorder - B Criteria Scale; ITQ, International Trauma Questionnaire; BFI, Brief Fatigue Inventory; FIS, Fatigue Impact Scale; MoCA, Montreal Cognitive Assessment; DSST, Digit Symbol Substitution Test (DSST1: number of correct symbols within 90 sec., DSST2: number of correct symbols from memory); TMT, Trail Making Test.

Over time (T1 to T3), female patients (ΔDSST2: Mdn=2.00, IQR: 0.00-3.00) as well as patients with a mild-moderate COVID-19 course (ΔDSST2: Mdn=2.00, IQR: 0-3.00) showed a greater improvement in memory performance (DSST2) than male patients (ΔDSST2: Mdn=0.00, IQR: -1.00-1.75, p=0.003, r=0.292) or those with a severe to critical acute course (ΔDSST2: Mdn=1.00, IQR: -1.00-2.00, p=0.028, r=-0.212). Post-COVID patients working within the healthcare sector improved from T1 to T3 slightly in their depression severity (ΔHADS-D_Depression_: Mdn=-1, IQR: -3-1) whereas non-healthcare-workers stayed at the same level (ΔHADS-D_Depression_: Mdn=0, IQR: -1-3, p=0.020, r=0.209). Furthermore, patients with a pre-existing mental impairment in the T1-T3 course showed an improvement in their fatigue severity (ΔBFI: Mdn=-0.41 IQR: -1.13-0.44), while patients without a pre-existing mental impairment worsened in their fatigue severity (ΔBFI: Mdn=0.33, IQR: -0.66-1.30, p=0.014, r=-0.227). TMT-A showed a significant group difference between healthcare-workers (ΔMdn=-4.84; IQR: -11.18-1.86) and non-healthcare-workers (ΔMdn=3.00; IQR: -10.70-9.34; p=0.035; r=0.203). Additionally, patients with a pre-existing psychological disease demonstrated a greater reduction in ITQ (ΔMdn=-4.00; IQR: -8.00-0.50) compared to those without a pre-existing psychological disease (ΔMdn=-1.00, IQR: -3.00-2.00, p=0.020, r=-0.215). For a complete overview of the conducted group analysis regarding the change from T1 to T3 see the corresponding tables (**Supplementary Tables A1**, **A3**, **A5**, **A7**, **A9**, **A11**, **A13**, **A15**, **A17**, **A19**, **A21**) in the **Supplementary Material**.

### 3.2 12 months after rehabilitation

At T4, 12.3% of patients had mild symptoms of depression (HADS-D_Depression_) and 26.7% showed symptoms of severe depression (**Supplementary Figure 5**). At T4, 17.1% of patients had mild symptoms and 21.0% had severe symptoms in anxiety (HADS-D_Anxiety_) (**Supplementary Figure 6**). 12 months after rehabilitation 21.4% patients showed symptoms of mild cognitive impairment (MoCA) (**Supplementary Figure 7**). 58.8% of patients showed moderate and 26.3% severe symptoms of fatigue (BFI) (**Supplementary Figure 8**).

From the beginning of inpatient rehabilitation (T1) to 12 months after rehabilitation discharge (T4), various improvements were achieved within the cognitive and mental health assessment (see **Table 3**). The HADS-D showed a significant improvement in the patients’ anxiety symptoms (HADS-D_Anxiety_: T1: Mdn=6.00, IQR: 4.00-11.00; T4: Mdn=5.00; IQR: 3.00-10.00; p=0.040) with a low effect size (r=-0.200). A significant change was observed in the ITQ score between T1 (Mdn=7.00; IQR: 3.00-13.00) and T4 (Mdn=4.00; IQR: 1.00-9.00; p<0.001) with a medium effect size (r=-0.396). Furthermore, the post-COVID patients showed a significant worsening of fatigue (BFI) (T1: Mdn=5.47, IQR: 4.22-6.58; T4: Mdn=6.00, IQR: 4.63-7.00; p=0.017) with a low effect size (r=0.224). The depressive symptoms (HADS-D_Depression_), SSD-12, FIS, and subjective mental health did not change significantly between T1 and T4 (p>0.05). Within the cognitive performance measures from T1 to T4, significant improvements in executive functions in the context of working memory and processing speed (DSST1: T1: Mdn= 46.00, IQR: 37.00-53.00; T4: Mdn=49.00, IQR: 37.00,75.00-58.25; p<0.001) with moderate effect size (r=0.364), as well as recall performance (DSST2: T1: Mdn=5.00, IQR: 2.00-6.25; T4: Mdn=6.00, IQR: 4.00-8.00; p<0.001) with a medium effect size (r=0.485) were determined. TMT-A showed a significant change from T1 (Mdn=34.35, IQR: 27.00-44.47) to T4 (Mdn=29.19, IQR: 24.10-41.21; p<0.001) with a medium effect size (r=-0.374). Similarly, TMT-B changed significantly from T1 (Mdn=71.03, IQR: 57.94-88.32) to T4 (Mdn=65.90, IQR: 54.93-86.43; p=0.027) with low effect size (r=-0.223).

**Table 3:** Differences in neuropsychological health and fatigue between T1 and T4.

	N	T1			T4			z	p	r
		Min	Max	Median (IQR)	Min	Max	Median (IQR)			
HADS-D_Depression_	105	0.00	19.00	7.00 (4.00 – 11.00)	0.00	20.00	6.00 (4.00 – 11.00)	-0.907	0.365	-0.089
HADS-D_Anxiety_	105	0.00	18.00	6.00 (4.00 – 11.00)	0.00	19.00	5.00 (3.00 – 10.00)	-2.054	0.040	-0.200
SSD-12	112	1.00	43.00	25.00 (17.00 – 31.00)	0.00	45.00	23.00 (16.25 – 31.00)	-1.167	0.243	-0.110
ITQ	112	0.00	22.00	7.00 (3.00 – 13.00)	0.00	24.00	4.00 (1.00 – 9.00)	-4.195	<0.001	-0.396
BFI	114	0.67	8.89	5.47 (4.22 – 6.58)	0.00	9.33	6.00 (4.63 – 7.00)	2.391	0.017	0.224
FIS	112	0.00	143.00	95.50 (72.50 – 11.75)	0.00	159.00	97.00 (73.25 – 117.00)	0,520	0.603	0.049
MoCA	98	22.00	30.00	27.00 (25.00 – 28.00)	19.00	30.00	27.00 (26.00 – 28.00)	1.149	0.250	0.116
DSST1	98	11.00	84.00	46.00 (37.00 – 53.00)	13.00	88.00	49.00 (37.75 – 58.25)	3.600	<0.001	0.364
DSST2	98	0.00	9.00	5.00 (2.00 – 6.25)	0.00	9.00	6.00 (4.00 – 8.00)	4.803	<0.001	0.485
TMT-A	98	13.80	130.00	34.35 (27.00 – 44.47)	14.90	180.00	29.19 (24.10 – 41.21)	-3.703	<0.001	-0.374
TMT-B	98	31.40	300.00	71.03 57.94 – 88.32)	24.10	221.00	65.90 (54.93 – 86.43)	-2.206	0,027	-0.223
Subj. mental health	113	0.82	9.36	5.36 (4.54-6.63)	0.36	9.82	5.27 (4.36-6.63)	0.389	0.697	0.037

HADS-D, German Hospital Anxiety and Depression Scale; SSD-12, Somatic Symptom Disorder - B Criteria Scale; ITQ, International Trauma Questionnaire; BFI, Brief Fatigue Inventory; FIS, Fatigue Impact Scale; MoCA, Montreal Cognitive Assessment; DSST, Digit Symbol Substitution Test (DSST1: number of correct symbols within 90 sec., DSST2: number of correct symbols from memory), TMT, Trail Making Test.

Younger patients showed a greater improvement from T1 to T4 in cognitive performance (ΔDSST1: Mdn=5.00, IQR: 0.50-12.50) than older patients (ΔDSST1: Mdn=2.00, IQR: -3.00-6.50, p=0.025, r=-0.227), and in terms of sex, female patients were found to improve to a greater extend at T4 (ΔDSST2: Mdn=2.00, IQR: 0.00-3.00) than male patients (ΔDSST2: Mdn=1.00, IQR: -0.25-2.00, p=0.003, r=0.292). Over the course of 12 months, patients with a mild-moderate COVID-19 course showed greater improvements in subjectively perceived mental health (Δ: Mdn=0.14, IQR: -0.66-1.18) as well as in cognitive performance (ΔDSST2: Mdn=2.00, IQR: 0.00-3.00) than patients with severe-critical COVID-19 (Δsubj. mental health: Mdn=-0.64 IQR: -1.55-0.45, p=0.026, r=-0.209; ΔDSST2: Mdn=1.00, IQR: -1.00-2.00, p=0.043, r=-0.204). At T4, there was a significant difference in the change in fatigue severity (BFI score) with regard to socioeconomic status. Post-COVID patients with a medium socioeconomic status worsened to a greater extent (ΔSES: Mdn=0.88, IQR: 0.00-1.27) than post-COVID patients with a high socioeconomic status (ΔSES: Mdn=0.27, IQR: -0.88-1.08, p=0.033, r=-0.201). Patients with and without a pre-existing mental impairment also differed in terms of the change in fatigue severity from T1 to T4. Patients with a pre-existing mental impairment improved significantly in terms of their fatigue severity (ΔBFI: Mdn=-0.55, IQR: -1.38-0.47) from T1 to T4. In contrast, patients without a pre-existing mental impairment showed a significant deterioration in the BFI score over the 12-month follow-up (ΔBFI: Mdn=0.66, IQR: -0.77-1.22, p<0.001, r=-0.320). A significant group difference was observed in the TMT-B test between patient with pre-existing cardiovascular disease (ΔMdn=0.46; IQR: -13.40-16.08) and without cardiovascular disease (ΔMdn=-8.39; IQR: -22.20-4.30p=0.026; r=0.225). Additionally, patients with a psychological disease demonstrated a greater reduction in FIS score (ΔMdn=-10.00; IQR: -20.50-8.00) compared to those without a psychological disease (ΔMdn=2.00, IQR: -10.00-18.00, p=0.026, r=-0.210). Furthermore, patients with a neuro-sensory disease showed less improvement the TMT-B test (ΔMdn=3.62, IQR: -13.22-25.50) compared to those without neuro-sensory disease (ΔMdn=-7.41, IQR: -21.92-4.83, p=0.022, r=0.232). For a complete overview of the conducted group analysis regarding the change from T1 to T4 see the corresponding tables (**Supplementary Tables A2**, **A4**, **A6**, **A8**, **A10**, **A12**, **A14**, **A16**, **A18**, **A20**, **A22**) in the **Supplementary Material**.

## 4 Discussion

This study is one of the first studies to assess mental health and cognitive function of post-COVID patients six and 12 months after inpatients post-COVID rehabilitation. In general, cognitive function improved after rehabilitation and could be maintained during follow-up. The results of mental health are heterogeneous and depend on the parameters measured.

### 4.1 Prevalence of psychological symptoms, fatigue, and cognitive impairment

The prevalence of depression and anxiety symptoms in our study showed slight improvement over time. However, at T4, 39.1% of patients still exhibited mild to severe symptoms of depression, and 38.1% had mild to severe symptoms of anxiety. Compared to previous studies, the prevalence of symptoms of depression and anxiety are higher (19, 64). Fatigue remained a persistent and significant issue in our cohort, with 61.0% of patients reporting moderate and 23.7% severe fatigue at T1. These levels remained relatively stable at T3 and T4, indicating that fatigue is a long-lasting symptom for many post-COVID-19 patients in our study. These findings are consistent with other research, which reported prevalence rates of fatigue between 44-60% up to 24 months post-infection (16, 19). Van Wambeke et al. (18) specifically noted that fatigue was the most common symptom, present in more than 90% of post-COVID patients over a two-year follow-up period. This emphasizes the need for targeted interventions to manage fatigue more effectively, as it significantly impacts the quality of life and overall recovery in post-COVID-19 patients. The prevalence of mild cognitive impairment in our study showed a mixed trend, initially increasing from 25.9% at T1 to 30.6% at T3, before decreasing to 19.4% at T4. This suggests that cognitive recovery may take longer and varies among individuals. Our findings align with other research showing that cognitive impairment prevalence can be as high as 50% four months after acute infection (65). Over time, the prevalence tends to decrease, however 24-29% of patients still exhibit cognitive deficits nine months post-infection (66, 67). Long-term studies also indicate that around 20% of patients continue to experience cognitive impairments up to 24 months after the acute infection (64). The existing impairments have a crucial impact on the patient’s return to social and professional life (68, 69). Therefore, long-term treatment strategies are needed to address the high and complex psychological symptom burden of post-COVID patients in an adequate manner.

### 4.2 Changes in mental and cognitive health and fatigue over time

The results of our previous published paper show, that directly after rehabilitation the scores of HADS-D_Depression_ and HADS-D_Anxiety_ improved significantly (33). This is in line with other studies (30, 31) and review-analyses (34, 35). The results suggest a good effectiveness of the implemented multidisciplinary inpatient rehabilitation program even if the missing control group prohibits causal statements. The current investigation demonstrates that six to 12 months after rehabilitation discharge the scores of HADS-D_Depression_ and HADS-D_Anxiety_ are approaching the baseline level (T1). Similar to depression and anxiety, fatigue severity (measured by BFI) is improving after rehabilitation (33). However, the improvements in fatigue are not maintained at T3, and in fact worsen significantly at 12 months follow-up. The long course of the disease with diverse symptoms, the everyday restrictions experienced as a result of the disease, the uncertainty about the further course of disease and recovery, existential worries regarding the return to work (including financial concerns), the patient’s social life and if applicable also the working environment can play a decisive role here, and may even increase psychological stress (70, 71). Furthermore, it is known, that reduced physical capacity is associated with decreased psychological symptoms, a relationship observed in both general contexts and specific conditions such as lung diseases (72, 73). The COVID-19 pandemic has further illuminated the connection between physical and mental health. According to a recent study, post-COVID patients with physical impairments were associated with more cognitive dysfunction (74). Reduced physical capacity can trigger several physiological mechanisms that contribute to increased psychological symptoms in post-COVID patients. These include disruptions in neurotransmitter regulation, impairment of neuroplasticity, immune system dysregulation, microvascular injury, and exacerbation of inflammatory processes (75–77). During rehabilitation, the patients received intensive psychological care. The absence of ongoing psychological support after rehabilitation discharge, combined with the return to everyday life and work, may have contributed to the observed worsening of symptoms. Further, if return to work is not possible due to continued presence of post-COVID condition and the associated reduced work ability, patients may experience financial strain which also negatively impacts their mental health in the long-term. Our findings suggest that patients with medium SES experienced a greater worsening in their BFI scores compared to those with high SES from T1 to T4. This underscores how socioeconomic factors significantly influence the mental health trajectory of post-COVID patients over time. In addition, reduced social activities and the loss of social support as the disease progresses could also be reasons for worsening mental health (78, 79). The results measured by standardised and validated questionnaires (HADS-D and BFI) are supported by the subjective perceived mental health, assessed by a self-generated questionnaire. After rehabilitation, the post-COVID patients perceived an improvement in their mental health (see Müller et al. (33)), but this deteriorated again after six and 12 months analogously to the scores for depression and fatigue. Based on the results, the ITQ scores show significant improvements six and 12 months after rehabilitation, indicating a reduction in post-traumatic stress symptoms among post-COVID patients. This finding aligns with another study that reported a gradual decrease in trauma-related symptoms at a 3- and 6-month follow-up (22). In contrast, the SSD-12 scores do not show significant improvements and remain at a high level, suggesting persistent somatic symptoms. This is consistent with findings from other studies that highlight the ongoing presence of somatic complaints in post-COVID patients and demonstrate an association between baseline SSD-12 scores and persistent symptoms at follow-up (80, 81).

Contrary to mental health, the results of the cognitive function are more positive. The significant improvement in processing speed (DSST1) and memory performance (DSST2), ability to switch between different cognitive tasks (sequencing and attention, TMT-A), and cognitive flexibility (TMT-B) after rehabilitation discharge (T2) could be maintained over six to 12 months follow-up. The change in the DSST1 score even reaches the MCID of 3-5 points. These improvements are substantial compared to normative data, suggesting that while patients still show some impairment compared to healthy controls, their cognitive function has markedly improved over time (82). Comparative studies have reported analogous findings. A recent study showed that the results of the TMT were significantly higher in the post-COVID-19 group compared to healthy people (83). However, the significant improvement in MoCA score at T2 couldn’t be maintained during the 12 months follow-up period. This underlines current findings of Lynch et al. (84), revealing a lack of sensitivity of the MoCA regarding the cognitive complaints of post-COVID patients in their study.

The revealed group differences point out that the pre-existing mental impairment influences the course of fatigue-severity in the mid- and long-term. According to the assessed BFI score, patients with pre-existing mental impairment showed better BFI scores at T3 and T4 compared to T1. Probably these patients get already psychological support before rehabilitation and the transition from rehabilitation to aftercare is more fluent. Since patients without pre-existing mental impairment worsen within their fatigue severity during follow-up, more medical support is needed to counteract this increase. Rehabilitation providers must take this into account and should also encourage patients without history of mental impairment to seek ongoing psychological or medical treatment after rehabilitation to account for their long-lasting fatigue complaints. Regarding cognitive function over time, female post-COVID patients, younger post-COVID patients and patients with mild-moderate COVID-19 showed a higher improvement in cognitive function (DSST2) six and 12 months after rehabilitation. Nevertheless, male post-COVID patients, older post-COVID patients and patients with severe-critical COVID-19 also improved within follow-up regarding cognitive function although not in the same extent. Further, the socioeconomic status influences the course of fatigue severity since patients with a medium SES worsened to a greater extent than patients with a high SES. Financial reserves, better physical and mental working conditions and higher (health-related) human capital (educational level) possibly relieve the patients of the burden of disease in a greater extend. There is research highlighting the influence of a higher SES on health behaviour, morbidity, and access to medical services (85, 86). Therefore, it is necessary to recognize and address the socio-economic factors in health research approaches that impact individuals’ quality of life. However, even the post-COVID patients with a high SES worsen in their fatigue severity 12 months after rehabilitation discharge.

### 4.3 Recommendations for improving rehabilitation management

General, the results of the follow-up timepoints illustrate the need of adequate and seamless aftercare strategies to maintain the improved mental health and cognitive function after inpatient rehabilitation. The long course of disease in some cases over several years is an additional psychological burden for the post-COVID patients. In addition, post-COVID patients are exposed to stigmatization as the disease is not always taken seriously (87). Stigmatization and stereotyping also affects the course of disease in a negative way and even prevents patients from seeking for psychological assistance (88). Educating the society and health care providers about post-COVID syndrome and providing patients long-term psychological support can contribute to improve mental health. Individual resources and self-management strategies should also be strengthened by psychological interventions within rehabilitation and during the aftercare process. The ability to perceive one’s own health status is an essential component of the implementation of self-management techniques, such as the PACING technique. It is crucial for patients to be able to accurately assess their individual and daily mental, cognitive, and physical resources to maintain their daily activities and tasks within their energy limits. Further, the German S1 guideline Long-/Post-COVID emphasizes the positive effects of sport and exercise programs on mental health and fatigue (2). Thus, patients should be encouraged to participate in exercising in an outpatient group e.g., in rehabilitation sport clubs or other adequate exercise programs after rehabilitation discharge. One exception to this is the group of post-COVID patients diagnosed with ME/CFS. The current guideline does not recommend exercise therapy for ME/CFS patients (89). Nevertheless, Gloeckl et al. (90) propose the implementation of person-oriented exercise therapy, which should be tailored to the severity of post-exertional malaise and the individual’s needs, limitations, and tolerance to exercising. At last, multimodal therapeutic approaches must be utilized in aftercare. Digital and telemedicine solutions for aftercare can be a promising method to improve symptom management in post-COVID patients. Studies highlight that telehealth platforms, such as video consultations and remote monitoring, alongside health apps and teletherapy, provide continuous support and effective symptom control, facilitating early intervention and personalized treatment adjustments (91–93). Reviews further underscore the efficacy of telerehabilitation in managing COVID-19 patients (94). This may enable more specific and individualized measures for the effective management of exercise-related rehabilitation and aftercare processes.

### 4.4 Limitations

There are some limitations which should be considered when interpreting the results. Due to ethical aspects, it was not possible to implement a control group within our study. Thus, the current findings can’t be fully attributed to inpatient rehabilitation, but they may also be a result of natural recovery. But we agree with Hayden et al. (30) that improvements after rehabilitation are more likely to be a result of rehabilitation than from natural recovery. Further, the study sample is quite small and the selective study population with work-related SARS-CoV-2 infection attending an inpatient rehabilitation at only one clinic has to be mentioned as a potential source of bias in participant recruitment and affecting the representativeness of the study population. However, the gained results seem suitable for comparisons with further post-COVID research data. 70% of included patients are working within the healthcare-sector. Healthcare-workers may have special professional circumstances, in particular during the COVID-19 pandemic. They were reporting higher suicidal ideation, increased stress, and decreased quality of life than other professionals (95). Further, the sample size of the different subgroups sex, age, COVID-19 severity, profession, socioeconomic status, and pre-existing diseases were not well balanced. More women than men were included in the current study sample, which is a result of the high amount of women working within the healthcare sector (96). Moreover, some of the neuropsychological instruments used, such as the MoCA, are screening measures rather than comprehensive neuropsychological assessments (97). This should be considered when interpreting the cognitive outcomes, as screening tools have limitations in detecting mild cognitive impairments (98). While the study follows patients for 12 months, longer follow-up periods (e.g., 24 months or more) would provide a more comprehensive understanding of the long-term effects of rehabilitation. A one-year observation period allows for insights into recovery trajectories; however, post-COVID symptoms have been reported to persist beyond this timeframe in some individuals (99). Extending the follow-up period could help determine whether improvements observed at 12 months remain stable, continue to progress, or potentially regress over time. Additionally, long-term data could provide valuable insights into the need for ongoing interventions. At last, the conducted analyses and group comparisons indicate that other multivariate and intra-individual analysis methods can provide further important insights to account for the complex associations and highly individual post-COVID courses. The mentioned limitations must be considered in comparison with findings from other studies in the context of COVID-19 rehabilitation.

## 5 Conclusion

The current analysis is one of the first, examining the course of post-COVID patients’ mental health and cognitive performance up to 12 months after inpatient rehabilitation. Previous results indicated an improvement in depressive symptoms and anxiety, fatigue severity and cognitive function direct after inpatient rehabilitation. The missing improvements in our study six- and 12-months follow-up in depression, somatic symptom disorders, and fatigue indicate the importance of the aftercare process and the implementation of adequate therapeutic interventions such as psychological support and strengthen self-management skills. The results could also identify factors influencing the symptom recovery in the mid- and long-term. Nevertheless, future research is needed to evaluate how post-COVID patients after inpatient rehabilitation can be best accompanied and supported in the long course of their disease.

## Data Availability

The raw data supporting the conclusions of this article will be made available from the corresponding author upon justified request.
