# Supplementary material for: Course of neuropsychological health in post-COVID patients differs 6 and 12 months after inpatient rehabilitation
Source: Front Psychiatry. 2025 Apr 25;16:1460097. doi: 10.3389/fpsyt.2025.1460097 (PMC12062137; doi:10.3389/fpsyt.2025.1460097)
Supplement: Supplementary file 1 [file SupplementaryFile1.docx]

**Supplementary Material**


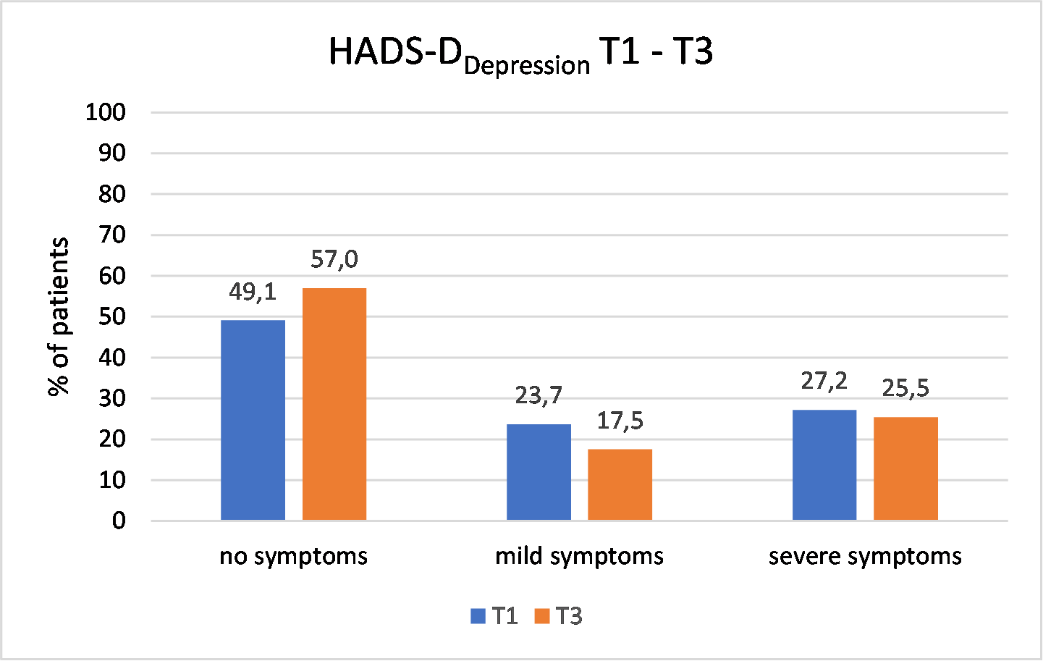


**Supplementary Figure 1.** Classification of depressive symptoms at T1 (blue) and T3 (orange) according to the HADS-D_Depression_ score. HADS-D - German Hospital Anxiety and Depression Scale.


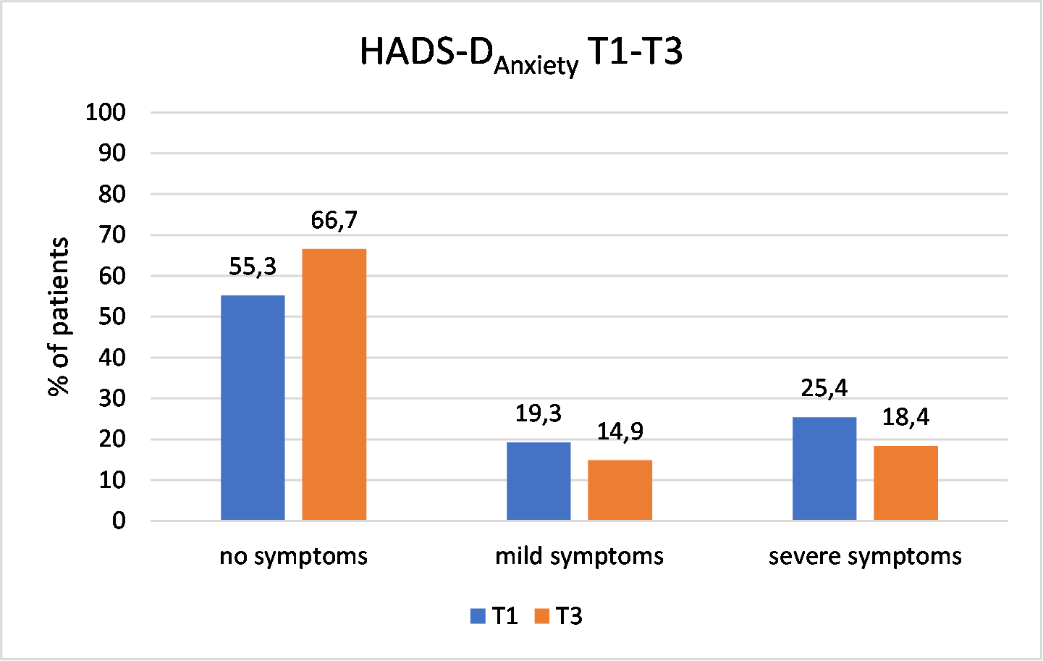


**Supplementary Figure 2.** Classification of symptoms of anxiety at T1 (blue) and T3 (orange) according to the HADS-D_Anxiety_ score. HADS-D - German Hospital Anxiety and Depression Scale.


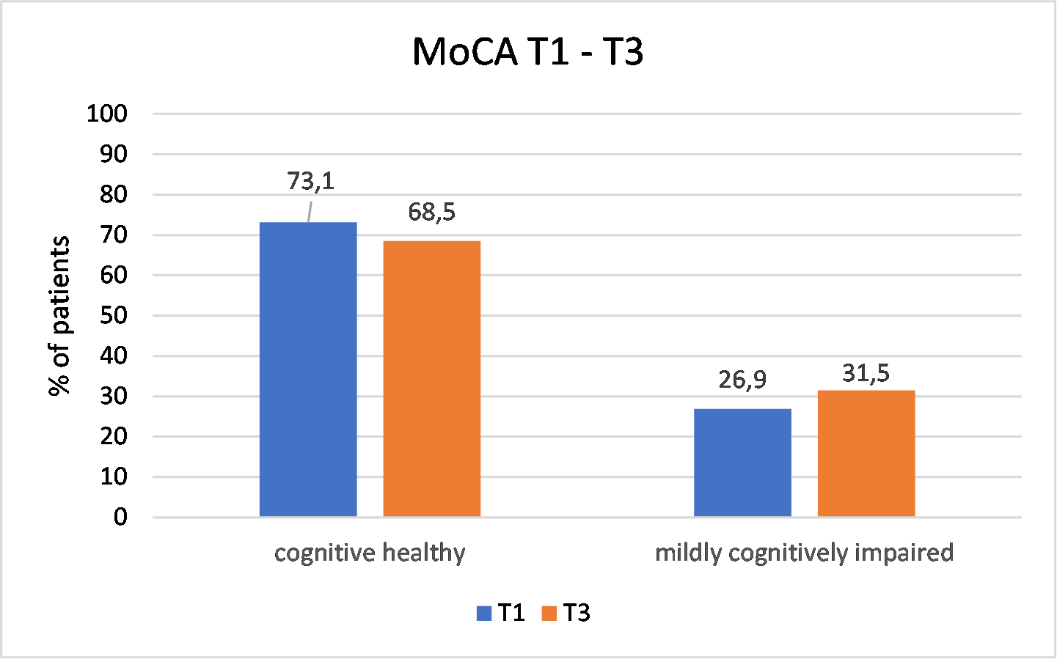


**Supplementary Figure 3.** Classification of cognitive impairment at T1 (blue) and T3 (orange) according to the MoCA score. MoCA - Montreal Cognitive Assessment.


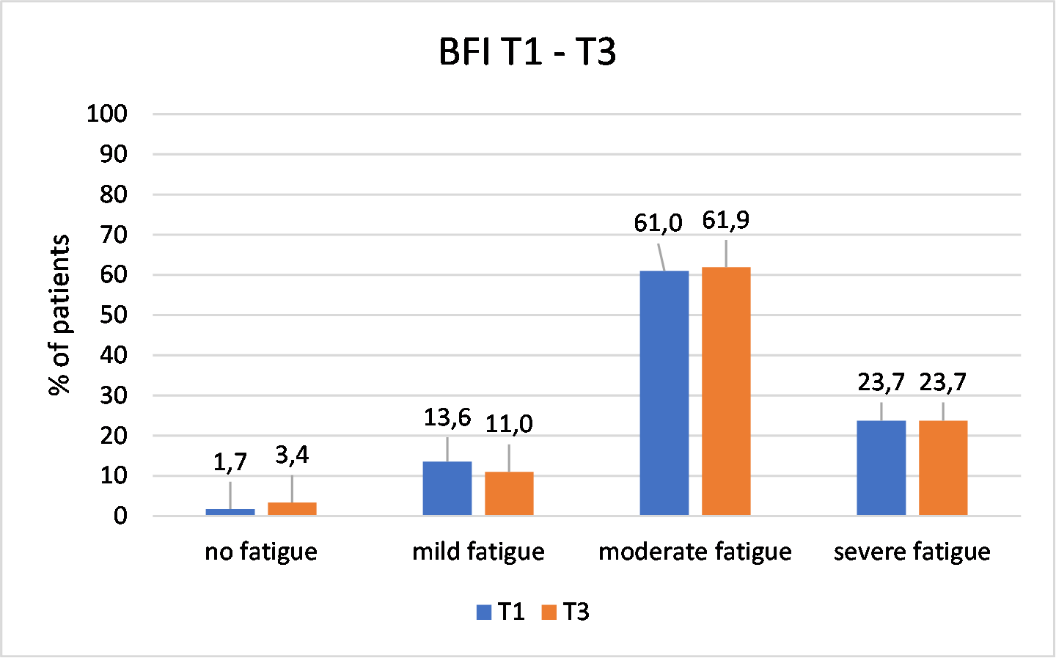


**Supplementary Figure 4.** Classification of fatigue severity at T1 (blue) and T3 (orange) according to the BFI score. BFI - Brief Fatigue Inventory.


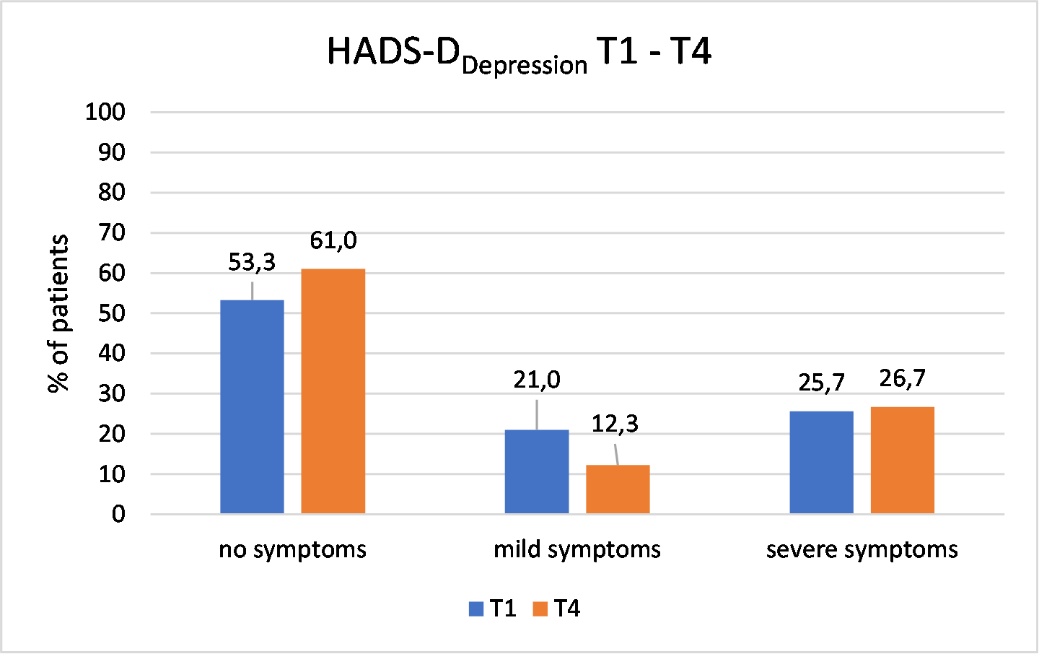


**Supplementary Figure 5.** Classification of symptoms of depression at T1 (blue) and T4 (orange) according to the HADS-D_Depression_ score. HADS-D - German Hospital Anxiety and Depression Scale.


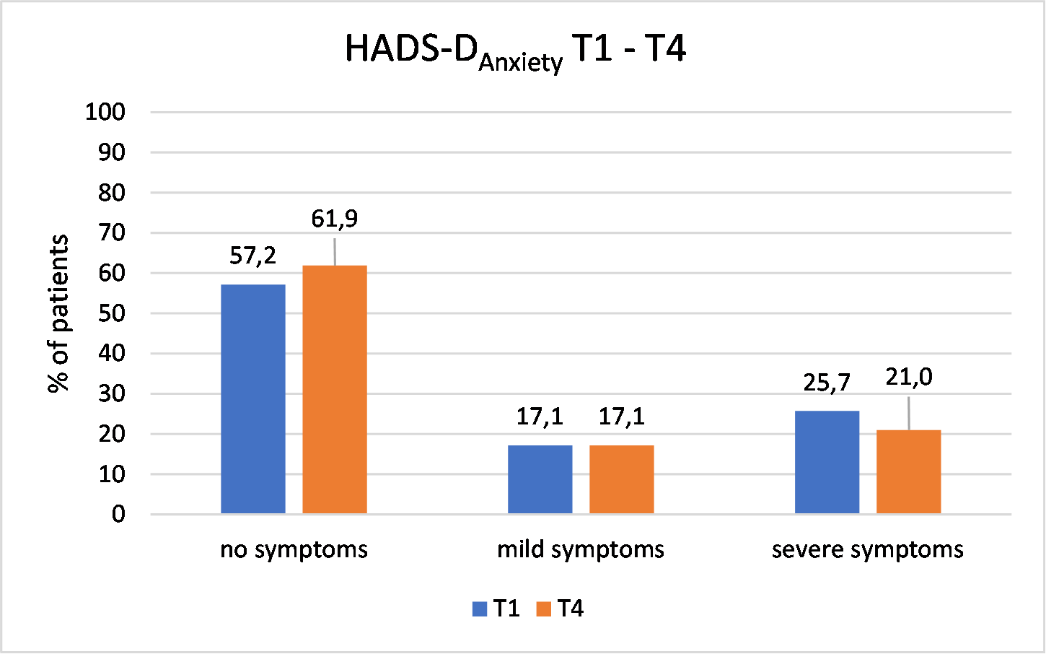


**Supplementary Figure 6.** Classification of symptoms of anxiety at T1 (blue) and T4 (orange) according to the HADS-D_Anxiety_ score. HADS-D - German Hospital Anxiety and Depression Scale.


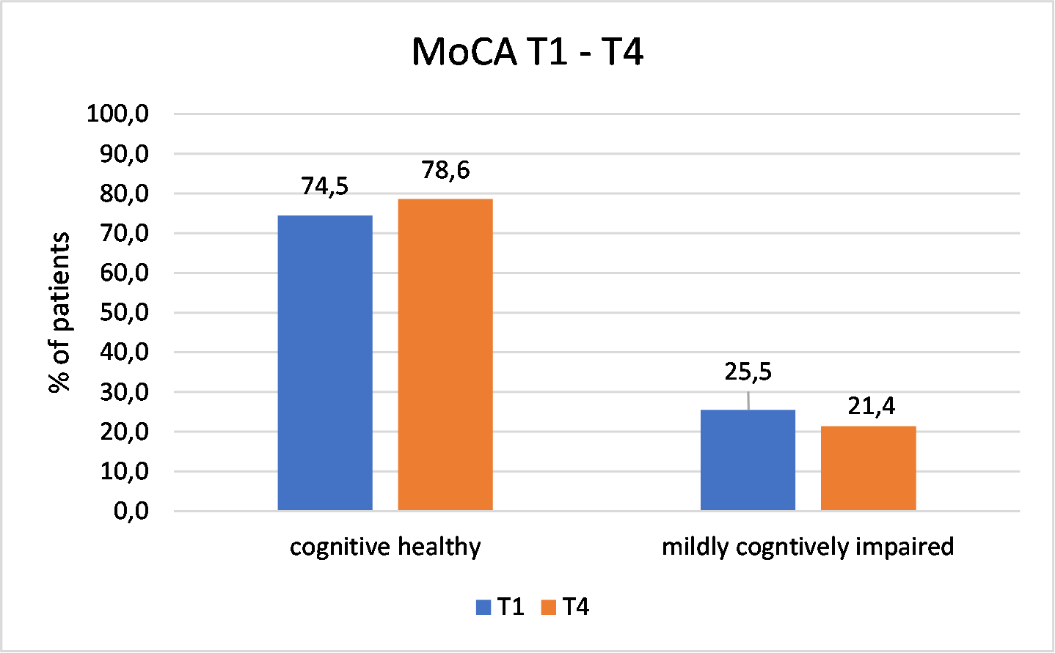


**Supplementary Figure 7.** Classification of cognitive impairment at T1 (blue) and T4 (orange) according to the MoCA score. MoCA - Montreal Cognitive Assessment.


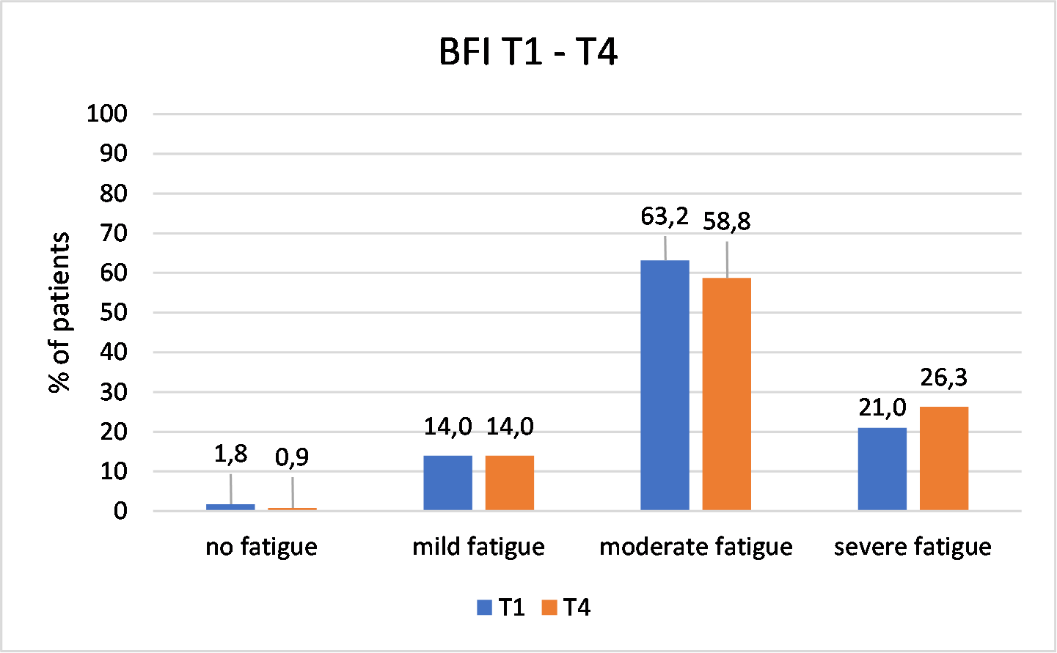


**Supplementary Figure 8.** Classification of fatigue severity at T1 (blue) and T4 (orange) according to the BFI score. BFI - Brief Fatigue Inventory.

Group analysis regarding the change of psychological parameters, fatigue, and cognitive parameters from T1 to T3 and T1 to T4.

| **Table A1:** Groupwise comparison of psychological parameters, fatigue, and cognitive parameters of male and female post-COVID patients between timepoints T1 and T3. | | | | | | | | | | | |
| --- | --- | --- | --- | --- | --- | --- | --- | --- | --- | --- | --- |
|  | **Sex** | | | | | | | | **Between-group** | | |
|  | **Male** | | | | **Female** | | | | **difference** | | |
|  | **N** | **T1**  **Median**  **(IQR)** | **T3**  **Median**  **(IQR)** | **Δ** | **N** | **T1**  **Median**  **(IQR)** | **T3**  **Median**  **(IQR)** | **Δ** | **z** | **p** | **r** |
| HADS-D_Depression_ | 29 | 8.00 (4.50 – 10.50) | 5.00 (2.50 – 8.00) | -1.00 (-3.50-0.00) | 85 | 7.00 (4.00 – 11.00) | 7.00 (3.00 – 11.00) | 0.00 (-2.00-2.00) | 1.713 | 0.087 | 0.160 |
| HADS-D_Anxiety_ | 29 | 5.00 (3.00 – 10.50) | 4.00 (1.50 – 7.00) | -2.00 (-3.00-0.50) | 85 | 7.00 (4.00 – 11.00) | 7.00 (4.00 – 10.00) | 0.00 (-2.50-2.00) | 1.393 | 0.164 | 0.131 |
| SSD-12 | 29 | 25.00 (15.00 – 33.00) | 22.00 (16.00 – 30.50) | -2.00 (-9.00-2.50) | 89 | 25.00 (19.50 – 31.50) | 24.00 (18.00 – 33.50) | 0.00 (-5.00-3.50) | 0.926 | 0.354 | 0.085 |
| ITQ | 28 | 6.50 (2.25 – 11.75) | 3.00 (1.00 – 11.50) | -1.50 (-5.00-1.75) | 89 | 7.00 (3.00 – 14.00) | 6.00 (2.00 – 10.50) | -1.00 (-4.00-2.00) | 0.304 | 0.761 | 0.028 |
| BFI | 29 | 5.22 (3.89 – 6.67) | 5.78 (4.56 – 6.44) | 0.00 (-0.88-1.05) | 89 | 5.56 (4.72 – 6.83) | 6.00 (4.89 – 7.11) | 0.22 (-0.66-1.16) | 0.566 | 0.571 | 0.052 |
| FIS | 28 | 83.50 (61.25 – 111.50) | 79.50 (60.75 – 103.50) | 1.50 (-15.25-9.50) | 89 | 99.00 (76.00 – 115.00) | 99.00 (72.00 – 115.00) | 0.00 (-11.00-13.50) | 0.435 | 0.664 | 0.040 |
| MoCA | 28 | 27.00 (25.00-28.75) | 27.00 (24.25-28-75) | 0.00 (-2.75-1.00) | 80 | 27.00 (25.25-28.00) | 27.00 (25.00-28.00) | 0.00 (-2.00-1.00) | 0.099 | 0.921 | 0.010 |
| DSST_1 | 28 | 41.00 (35.25-48.50) | 46.50 (35.25-52.00) | 2.50 (-3.25-7.75) | 79 | 48.00 (39.00-54.00) | 52.00 (45.00-60.00) | 5.00 (-1.00-9.00) | 0.926 | 0.354 | 0.090 |
| DSST_2 | 28 | 5.50 (4.00-7.00) | 7.00 83.00-8.009 | 0.00 (-1.00-1.75) | 79 | 4.00 (2.00-6.00) | 6.00 (4.00-8.00) | 2.00 (0.00-3.00) | 3.016 | 0.003 | 0.292 |
| TMT-A | 28 | 29.74 (26.25 – 39.77) | 29.70 (24.30 – 38.98) | -0.51 (-5.34-6.56) | 80 | 35.00 (28.03 – 45.40) | 31.00 (24.80 – 37.72) | -5.10 (-12.28-3.00) | -1.917 | 0.055 | -0.185 |
| TMT-B | 28 | 69.13 (54.57 – 93.25) | 70.59 (55.00 – 87.25) | -7.39 (-15.50-19.44) | 78 | 73.55 (60.99 – 88.32) | 69.10 (51.83 – 80.38) | -6.04 (-20.29-4.10) | -1.014 | 0.311 | -0.099 |
| Subj. mental health | 29 | 5.45 (4.59 – 7.00) | 5.18 (4.41 – 6.86) | 0.00 (-0.63-1.00) | 89 | 5.18 (4.32 – 6.23) | 5.09 (4.18 – 6.50) | 0.00 (-0.77-0.63) | -0.356 | 0.721 | -0.033 |
| IQR - interquartile range, HADS-D - German Hospital Anxiety and Depression Scale, SSD-12 - Somatic Symptom Disorder - B Criteria Scale, ITQ - International Trauma Questionnaire, BFI - Brief Fatigue Inventory, FIS - Fatigue Impact Scale, MoCA - Montreal Cognitive Assessment, DSST - Digit Symbol Substitution Test (DSST_1: number of correct symbols within 90 sec., DSST_2: number of correct symbols from memory), TMT - Trail Making Test. | | | | | | | | | | | |

| **Table A2:** Groupwise comparison of psychological parameters, fatigue, and cognitive parameters of male and female post-COVID patients between timepoints T1 and T4. | | | | | | | | | | | |
| --- | --- | --- | --- | --- | --- | --- | --- | --- | --- | --- | --- |
|  | **Sex** | | | | | | | | **Between-group** | | |
|  | **Male** | | | | **Female** | | | | **difference** | | |
|  | **N** | **T1**  **Median**  **(IQR)** | **T4**  **Median**  **(IQR)** | **Δ** | **N** | **T1**  **Median**  **(IQR)** | **T4**  **Median**  **(IQR)** | **Δ** | **z** | **p** | **r** |
| HADS-D_Depression_ | 26 | 7.50 (3.75 – 10.25) | 5.50 (2.75 – 8.25) | -1.00 (-3.25-0.25) | 79 | 7.00 (4.00 – 11.00) | 6.00 (4.00 – 11.00) | 0.00 (-2.00-2.00) | 1.517 | 0.129 | 0.148 |
| HADS-D_Anxiety_ | 26 | 5.50 (3.00 – 11.00) | 4.50 (2.75 – 9.00) | -1.50 (-4.00-1.00) | 79 | 6.00 (4.00 – 11.00) | 6.00 (4.00 – 11.00) | -1.00 (-3.00-2.00) | 0.984 | 0.325 | 0.096 |
| SSD-12 | 28 | 24.50 (15.00 – 32.00) | 22.50 (17.25 – 29.75) | -4.00 (-8.00-3.75) | 84 | 25.00 (19.25 – 30.00) | 24.00 (16.00 – 32.00) | -1.00 (-7.00-7.00) | 1.500 | 0.134 | 0.142 |
| ITQ | 27 | 6.00 (2.00 – 11.00) | 3.00 (1.00 – 11.00) | -2.00 (-4.00-1.00) | 5 | 7.00 (3.00 – 14.00) | 4.00 (1.00 – 9.00) | -2.00 (-6.00-0.00) | -0.696 | 0.486 | -0.066 |
| BFI | 28 | 5.17 (3.89 – 6.61) | 5.83 (4.11 – 6.86) | 0.33 (-0.22-1.05) | 86 | 5.53 (4.67 – 6.58) | 6.11 (5.00 – 7.00) | 0.55 (-0.66-1.22) | 0.217 | 0.828 | 0.020 |
| FIS | 28 | 83.50 (61.25 – 109.25) | 87.50 (58.00 – 97.50) | -4.00 (-14.75-13.50) | 84 | 99.00 (75.50 – 114.00) | 103.50 (75.25 – 119.50) | 3.00 (-12.00-14.75) | 1.032 | 0.302 | 0.098 |
| MoCA | 26 | 27.00 (24.75-29.00) | 27.00 (26.00-29.00) | 0.00 (-1.00-2.00) | 72 | 27.00 (26.00-28.00) | 27.00 (26.00-28.00) | 0.00 (-1.00-1.00) | -0.631 | 0.528 | -0.064 |
| DSST_1 | 26 | 40.50 (32.75-49.00) | 44.00 (34.75-53.50) | 3.50 (-1.25-10.00) | 72 | 47.50 (39.25-53.75) | 50.50 (41.25-59.75) | 3.00 (-3.00-9.00) | -0.072 | 0.942 | -0.007 |
| DSST_2 | 26 | 5.50 83.75-7.00) | 6.00 (3.00-8.00) | 1.00 (-0.25-2.00) | 72 | 4.00 (2.00-6.00) | 6.00 (4.00-8.00) | 2.00 (0.00-9.00) | 2.178 | 0.029 | 0.220 |
| TMT-A | 26 | 32.31 (26.25 – 48.50) | 28.42 (24.92 – 37.71) | -3.63 (-7.55-2.45) | 72 | 34.92 (27.25 – 43.68) | 29.74 (24.00 – 42.13) | -5.68 (-10.85-1.14) | -0.941 | 0.346 | -0.095 |
| TMT-B | 26 | 67.53 (54.25 – 95.50) | 68.95 (55.93 – 102.25) | 1.90 (-10.53-16.86) | 72 | 72.12 (60.47 – 87.25) | 64.37 (54.42 – 85.82) | -7.37 (-21.63-5.94) | -1.859 | 0.063 | -0.188 |
| Subj. mental health | 28 | 5.59 (4.66 – 7.00) | 5.64 (4.86 – 6.91) | 0.27 (-0.95-1.00) | 85 | 5.27 (4.45 – 6.31) | 5.09 (4.27 – 6.64) | 0.00 (-1.00-1.00) | -0.133 | 0.894 | -0.013 |
| IQR - interquartile range, HADS-D - German Hospital Anxiety and Depression Scale, SSD-12 - Somatic Symptom Disorder - B Criteria Scale, ITQ - International Trauma Questionnaire, BFI - Brief Fatigue Inventory, FIS - Fatigue Impact Scale, MoCA - Montreal Cognitive Assessment, DSST - Digit Symbol Substitution Test (DSST_1: number of correct symbols within 90 sec., DSST_2: number of correct symbols from memory), TMT - Trail Making Test. | | | | | | | | | | | |

| **Table A3:** Groupwise comparison of psychological parameters, fatigue, and cognitive parameters of younger and older post-COVID patients between timepoints T1 and T3. | | | | | | | | | | | |
| --- | --- | --- | --- | --- | --- | --- | --- | --- | --- | --- | --- |
|  | **Age** | | | | | | | | **Between-group** | | |
|  | **Younger than 51 years** | | | | **At least 51 years** | | | | **difference** | | |
|  | **N** | **T1**  **Median**  **(IQR)** | **T3**  **Median**  **(IQR)** | **Δ** | **N** | **T1**  **Median**  **(IQR)** | **T3**  **Median**  **(IQR)** | **Δ** | **z** | **p** | **r** |
| HADS-D_Depression_ | 42 | 6.00 (3.75 – 10.00) | 6.00 (3.00 – 9.00) | 0.00 (-3.00-2.00) | 72 | 8.00 (4.00 – 11.00) | 7.00 (3.00 – 11.00) | -1.00 (-2.00-1.75) | -0.534 | 0.593 | -0.050 |
| HADS-D_Anxiety_ | 42 | 6.00 (3.00 – 10.00) | 5.50 (3.00 – 7.25) | 0.00 (-2.00-1.00) | 72 | 7.00 (4.00 – 11.00) | 7.00 (3.00 – 10.00) | -1.00 (-3.00-2.00) | -0.729 | 0.466 | -0.068 |
| SSD-12 | 44 | 25.50 (15.25 – 31.75) | 24.00 (17.25 – 33.00) | 0.00 (-8.50-6.75) | 74 | 24.00 (17.75 – 32.25) | 23.00 (17.75 – 33.00) | 0.00 (-5.50-2.25) | -0.482 | 0.630 | -0.044 |
| ITQ | 44 | 6.00 (2.00 – 12.75) | 4.50 (1.00 – 11.00) | -1.00 (-3.00-2.00) | 73 | 8.00 (3.00 – 14.00) | 6.00 (2.00 – 10.00) | -2.00 (-4.50-1.00) | -0.996 | 0.319 | -0.092 |
| BFI | 44 | 5.22 (4.14 – 6.92) | 5.61 (4.31 – 6.86) | 0.27 (-0.88-1.33) | 74 | 5.67 (4.86 – 6.67) | 5.89 (4.97 – 6.83) | 0.11 (-0.66-0.89) | -0.420 | 0.674 | -0.039 |
| FIS | 44 | 95.00 (75.50 – 116.25) | 93.00 (72.50 – 114.50) | 0.00 (-21.75-15.75) | 73 | 97.00 (68.00 – 111.50) | 94.00 (66.50 – 112.50) | -1.00 (-7.50-10.50) | -0.031 | 0.975 | -0.003 |
| MoCA | 40 | 27.50 (26.00-29.00) | 27.50 (26.00-29.00) | 0.00 (-1.75-1.00) | 68 | 27.00 (25.00-27.00) | 26.00 (24.00-28.00) | 0.00 (-2.00-1.00) | -0.607 | 0.544 | -0.058 |
| DSST_1 | 39 | 51.00 (42.00-58.00) | 56.00 (49.00-62.00) | 5.00 (-1.00-10.00) | 68 | 43.00 (36.00-50.00) | 47.50 (38.50-53.00) | 3.00 (-1.00-8.00) | -0.836 | 0.403 | -0.081 |
| DSST_2 | 39 | 6.00 (4.00-7.00) | 8.00 (6.00-9.00) | 1.00 (0.00-3.00) | 68 | 4.00 (2.00-6.00) | 5.00 (4.00-7.00) | 1.00 (-1.00-3.00) | -0.849 | 0.396 | -0.082 |
| TMT-A | 40 | 30.72 (23.94 – 37.35) | 25.40 (20.97 – 34.33) | -4.91 (-10.61-2.15) | 68 | 36.34 (28.35 – 47.00) | 32.00 (27.50 – 41.33) | -3.28 (-11.72-6.16) | 0.951 | 0.342 | 0.092 |
| TMT-B | 40 | 70.51 (54.42 – 82.75) | 54.40 (44.03 – 74.60) | -7.20 (-24.99-2.58) | 66 | 74.00 (63.75 – 91.26) | 73.73 (60.52 – 85.75) | -5.50 (-15.70-8.16) | 1.372 | 0.170 | 0.133 |
| Subj. mental health | 44 | 5.18 (4.45 – 6.25) | 5.09 (4.57 – 6.61 | 0.04 (-0.81-0.95) | 74 | 5.36 (4.38 – 6.59) | 5.00 (4.11 – 6.75) | 0.00 (-0.72-0.63) | -0.465 | 0.642 | -0.002 |
| IQR - interquartile range, HADS-D - German Hospital Anxiety and Depression Scale, SSD-12 - Somatic Symptom Disorder - B Criteria Scale, ITQ - International Trauma Questionnaire, BFI - Brief Fatigue Inventory, FIS - Fatigue Impact Scale, MoCA - Montreal Cognitive Assessment, DSST - Digit Symbol Substitution Test (DSST_1: number of correct symbols within 90 sec., DSST_2: number of correct symbols from memory), TMT - Trail Making Test. | | | | | | | | | | | |

| **Table A4:** Groupwise comparison of psychological parameters, fatigue, and cognitive parameters of younger and older post-COVID patients between timepoints T1 and T4. | | | | | | | | | | | |
| --- | --- | --- | --- | --- | --- | --- | --- | --- | --- | --- | --- |
|  | **Age** | | | | | | | | **Between-group** | | |
|  | **Younger than 51 years** | | | | **At least 51 years** | | | | **difference** | | |
|  | **N** | **T1**  **Median**  **(IQR)** | **T4**  **Median**  **(IQR)** | **Δ** | **N** | **T1**  **Median**  **(IQR)** | **T4**  **Median**  **(IQR)** | **Δ** | **z** | **p** | **r** |
| HADS-D_Depression_ | 37 | 6.00 (3.50 – 9.50) | 5.00 (3.00 – 8.00) | 0.00 (-4.00-2.00) | 68 | 7.50 (4.00 – 11.00) | 7.00 (4.00 – 12.00) | 0.00 (-2.00-2.00) | 0.920 | 0.358 | 0.090 |
| HADS-D_Anxiety_ | 37 | 5.00 (3.00 – 9.00) | 4.00 (2.50 – 7.00) | -1.00 (-3.50-7.25) | 68 | 7.00 (4.00 – 11.00) | 6.00 (4.00 – 10.75) | -1.00 (-3.00—2.00) | 0.856 | 0.392 | 0.084 |
| SSD-12 | 42 | 25.00 (15.00 – 29.25) | 24.00 (12.25 – 30.25) | -0.50 (-8.00-7.25) | 70 | 24.00 (17.75 – 32.00) | 23.00 (17.00 – 31.00) | -3.00 (-7.00-4.25) | -0.713 | 0.476 | -0.067 |
| ITQ | 43 | 6.00 (2.00 – 11.00) | 2.00 (0.00 – 9.00) | -1.00 (-5.00-1.00) | 69 | 8.00 (3.50 – 14.00) | 5.00 (1.00 – 11.00) | -2.00 (-5.00-0.00) | -0.618 | 0.536 | -0.058 |
| BFI | 43 | 5.22 (4.11 – 6.67) | 5.89 (4.56 – 6.78) | 0.66 (-0.66-1.22) | 71 | 5.56 (4.78 – 6.56) | 6.00 (4.67 – 7.00) | 0.33 (-0.66-1.11) | -0.420 | 0.674 | -0.039 |
| FIS | 43 | 95.00 (75.00 – 114.00) | 101.00 (76.00 – 120.00) | 6.00 (-19.00-18.00) | 69 | 97.00 (67.50 – 111.00) | 95.00 (69.50 – 114.00) | -2.00 (-11.50-13.50) | -0.401 | 0.688 | -0.038 |
| MoCA | 33 | 28.00 (26.00-29.00) | 28.00 (27.00-29.00) | 0.00 (-1.00-2.00) | 65 | 27.00 (25.00-27.00) | 27.00 (25.00-28.00) | 0.00 (-1.00-1.00) | -0.897 | 0.370 | -0.091 |
| DSST_1 | 33 | 51.00 (41.00-61.00) | 59.00 (48.00-68.00) | 5.00 (0.50-12.50) | 65 | 43.00 (36.00-50.00) | 46.00 (36.00-52.50) | 2.00 (-3.00-6.50) | -2.246 | 0.025 | -0.227 |
| DSST_2 | 33 | 6.00 (3.50-7.00) | 8.00 (5.00-9.00) | 1.00 (0.00-2.00) | 65 | 4.00 (2.00-6.00) | 5.00 (3.50-7.00) | 1.00 (0.00-3.00) | 0.160 | 0.873 | 0.016 |
| TMT-A | 33 | 30.00 (23.12 – 37.97) | 24.00 (20.40 – 28.30) | -6.84 (-10.07- -1.67) | 65 | 36.86 (28.91 – 47.50) | 32.00 (27.00 – 48.63) | -4.23 (-10.38-2.63) | 0.970 | 0.332 | 0.098 |
| TMT-B | 33 | 60.96 (52.60 – 77.46) | 56.00 (46.55 – 68.23) | -9.91 (-20.05-3.26) | 65 | 74.19 (63.50 – 93.72) | 71.80 (59.50 – 90.65) | -3.91 (-20.23-11.15) | 1.537 | 0.124 | 0.155 |
| Subj. mental health | 43 | 5.27 (4.45 – 6.36) | 5.18 (4.55 – 6.73) | -0.18 (-1.45-1.63) | 70 | 5.45 (4.55 – 6.75) | 5.32 (4.09 – 6.43) | 0.04 (-0.86-0.90) | -0.465 | 0.642 | -0.044 |
| IQR - interquartile range, HADS-D - German Hospital Anxiety and Depression Scale, SSD-12 - Somatic Symptom Disorder - B Criteria Scale, ITQ - International Trauma Questionnaire, BFI - Brief Fatigue Inventory, FIS - Fatigue Impact Scale, MoCA - Montreal Cognitive Assessment, DSST - Digit Symbol Substitution Test (DSST_1: number of correct symbols within 90 sec., DSST_2: number of correct symbols from memory), TMT - Trail Making Test. | | | | | | | | | | | |

| **Table A5:** Groupwise comparison of psychological parameters, fatigue, and cognitive parameters of patients with mild-moderate COVID-19 and severe-critical COVID-19 between timepoints T1 and T3. | | | | | | | | | | | |
| --- | --- | --- | --- | --- | --- | --- | --- | --- | --- | --- | --- |
|  | **Severity of acute COVID-19** | | | | | | | | **Between-group** | | |
|  | **Mild/moderate** | | | | **Severe/critical** | | | | **difference** | | |
|  | **N** | **T1**  **Median**  **(IQR)** | **T3**  **Median**  **(IQR)** | **Δ** | **N** | **T1**  **Median**  **(IQR)** | **T3**  **Median**  **(IQR)** | **Δ** | **z** | **p** | **r** |
| HADS-D_Depression_ | 79 | 7.00 (4.00 – 10.00) | 6.00 (3.00 – 10.00) | 0.00 (-3.00-2.00) | 35 | 9.00 (5.00 – 12.00) | 8.00 (4.00 – 12.00) | -1.00 (-2.00-2.00) | 0.522 | 0.602 | 0.050 |
| HADS-D_Anxiety_ | 79 | 6.00 (4.00 – 10.00) | 6.00 (3.00 – 8.00) | 0.00 (-3.00-2.00) | 35 | 8.00 (4.00 – 13.00) | 7.00 (4.00 – 12.00) | -1.00 (-3.00-1.00) | -0.068 | 0.946 | -0.006 |
| SSD-12 | 83 | 24.00 (16.00 – 32.00) | 22.00 (18.00 – 31.00) | 0.00 (-7.00-3.00) | 35 | 26.00 (21.00 – 33.00) | 28.00 (17.00 – 35.00) | 1.00 (-3.00-7.00) | 0.903 | 0.367 | 0.083 |
| ITQ | 82 | 6.00 (2.00 – 13.00) | 4.00 (1.00 – 10.00) | -1.00 (-5.00-1.00) | 35 | 9.00 (4.00 – 15.00) | 9.00 (5.00 – 15.00) | -1.00 (-3.00-3.00) | 0.964 | 0.335 | 0.089 |
| BFI | 83 | 5.56 (4.67 – 6.56) | 5.67 (4.44 – 6.67) | -0.11 (-0.88-1.11) | 35 | 5.50 (4.00 – 7.33) | 6.56 (5.56 – 7.33) | 0.44 (-0.55-1.55) | 1.412 | 0.158 | 0.130 |
| FIS | 82 | 88.50 (72.75 – 113.25) | 87.00 (65.00 – 113.00) | -1.00 (-17.75-13.25) | 35 | 101.00 (70.00 – 114.00) | 101.00 (88.00 – 118.00) | 2.00 (-8.00-17.00) | 1.322 | 0.186 | 0.122 |
| MoCA | 74 | 27.00 (25.00-29.00) | 27.00 (25.00-28.00) | 0.00 (-2.00-1.00) | 34 | 26.00 (24.50-27.00) | 26.00 (24.00-27.25) | 1.00 (-5.00-10.00) | 1.039 | 0.299 | 0.100 |
| DSST_1 | 73 | 48.00 (40.50-54.50) | 52.00 (45.00-58.00) | 5.00 (-1.00-9.00) | 34 | 40.50 (34.00-50.25) | 48.50 (34.00-55.25) | 2.25 (-1.00-9.25) | -0.245 | 0.807 | -0.024 |
| DSST_2 | 73 | 5.00 (3.00-6.50) | 7.00 (4.50-9.009 | 2.00 (0.00-3.00) | 34 | 4.00 (2.00-6.00) | 5.00 (3.00-7.00) | 1.00 (-1.00-2.00) | -2.193 | 0.028 | -0.212 |
| TMT-A | 74 | 32.00 (26.12 – 39.00) | 29.00 (23.00 – 36.91) | -3.78 (-9.89-3.69) | 34 | 38.88 (29.80 – 50.50) | 32.65 (25.00 – 43.88) | -4.37 (-14.87-3.81) | -0.731 | 0.465 | -0.070 |
| TMT-B | 74 | 70.51 (57.02 – 82.50) | 63.01 (50.95 – 77.25) | -7.20 (-16.40-4.42) | 32 | 82.47 (66.25 – 97.00) | 78.70 (62.20 – 99.90) | -5.26 (-19.77-16.02) | 0.616 | 0.538 | 0.060 |
| Subj. mental health | 83 | 5.27 (4.45 – 6.55) | 5.27 (4.36 – 6.81) | 0.00 (-72-0.81) | 35 | 5.09 (4.45 – 6.27) | 5.00 (3.73 – 5.45) | 0.00 (-0.23-0.70) | -1.043 | 0.297 | -0.096 |
| IQR - interquartile range, HADS-D - German Hospital Anxiety and Depression Scale, SSD-12 - Somatic Symptom Disorder - B Criteria Scale, ITQ - International Trauma Questionnaire, BFI - Brief Fatigue Inventory, FIS - Fatigue Impact Scale, MoCA - Montreal Cognitive Assessment, DSST - Digit Symbol Substitution Test (DSST_1: number of correct symbols within 90 sec., DSST_2: number of correct symbols from memory), TMT - Trail Making Test. | | | | | | | | | | | |

| **Table A6:** Groupwise comparison of psychological parameters, fatigue, and cognitive parameters of patients with mild-moderate COVID-19 and severe-critical COVID-19 between timepoints T1 and T4. | | | | | | | | | | | |
| --- | --- | --- | --- | --- | --- | --- | --- | --- | --- | --- | --- |
|  | **Severity of acute COVID-19** | | | | | | | | **Between-group** | | |
|  | **Mild/moderate** | | | | **Severe/critical** | | | | **difference** | | |
|  | **N** | **T1**  **Median**  **(IQR)** | **T4**  **Median**  **(IQR)** | **Δ** | **N** | **T1**  **Median**  **(IQR)** | **T4**  **Median**  **(IQR)** | **Δ** | **z** | **p** | **r** |
| HADS-D_Depression_ | 74 | 6.00 (3.75 – 9.00) | 5.50 (3.00 – 10.00) | 0.00 (-3.00-2.00) | 31 | 8.00 (4.00 – 12.00) | 8.00 (5.00 – 13.00) | 0.00 (-2.00-3.00) | 1.139 | 0.254 | 0.111 |
| HADS-D_Anxiety_ | 74 | 6.00 (4.00 – 10.25) | 5.00 (3.00 – 9.00) | -1.00 (3.25-1.00) | 31 | 8.00 (3.00 – 12.00) | 8.00 (5.00 – 11.00) | -1.00 (-2.00-4.00) | 1.468 | 0.142 | 0.143 |
| SSD-12 | 81 | 24.00 (16.00 – 30.50) | 22.00 (14.00 – 28.50) | -3.00 (-8.00-4.50) | 31 | 26.00 (21.00 – 32.00) | 30.00 (20.00 – 35.00) | 2.00 (-5.00-8.00) | 1.312 | 0.190 | 0.124 |
| ITQ | 80 | 6.00 (2.00 – 11.75) | 3.00 (1.00 – 7.00) | -2.00 (-5.00-0.00) | 32 | 9.00 (4.25 – 14.75) | 8.00 (1.25 – 14.75) | -2.00 (-6.00-3.00) | 0.271 | 0.786 | 0.026 |
| BFI | 82 | 5.56 (4.58 – 6.56) | 5.89 (4.42 – 6.69) | 0.33 (-0.66-1.11) | 32 | 5.44 (4.00 – 6.92) | 6.33 (5.25 – 7.08) | 0.55 (-0.27-1.41) | 1.423 | 0.155 | 0.133 |
| FIS | 81 | 89.00 (72.50 – 112.00) | 94.00 (69.50 – 116.50) | 0.00 (-15.50-14.00) | 31 | 102.00 (70.00 – 112.00) | 104.00 (89.00 – 117.00) | 2.00 (-8.00-17.00) | 1.275 | 0.202 | 0.121 |
| MoCA | 68 | 28.00 (26.00-29.00) | 28.00 (27.00-29.00) | 0.00 (-1.00-1.75) | 30 | 27.00 (25.00-27.00) | 27.00 (25.00-28.00) | 0.50 (-1.00-2.00) | 0.737 | 0.461 | 0.074 |
| DSST_1 | 68 | 47.00 (38.25-54.00) | 51.00 (43.00-60.00) | 3.50 (-2.75-10.00) | 30 | 41.00 (34.00-50.25) | 46.00 (34.75-53.75) | 2.50 (-3.00-6.25) | -0.255 | 0.799 | -0.026 |
| DSST_2 | 68 | 5.00 (2.25-6.00) | 6.50 (4.00-8.00) | 2.00 (0.00-3.00) | 30 | 4.50 (2.00-7.00) | 5.50 (3.00-7.00) | 1.00 (-1.00-2.00) | -2.023 | 0.043 | -0.204 |
| TMT-A | 68 | 32.14 (26.07 – 39.11) | 27.35 (22.20 – 34.87) | -4.64 (-9.97-1.45) | 30 | 38.88 (28.95 – 50.50) | 35.66 (27.00 – 50.29) | -4.74 (-11.67-4.41) | 0.035 | 0.972 | 0.004 |
| TMT-B | 68 | 68.68 (56.25 – 83.50) | 64.00 (53.22 – 86.39) | -4.84 (-15.39-6.15) | 30 | 79.50 (66.47 – 95.00) | 68.28 (58.89 – 87.06) | -8.07 (-21.30-8.45) | -0.624 | 0.532 | -0.063 |
| Subj. mental health | 82 | 5.36 (4.52 – 6.72) | 5.55 (4.52 – 7.00) | 0.13 (-0.65-1.18) | 31 | 5.45 (4.55 – 6.36) | 4.64 (3.82 – 5.73) | -0.63 (-1.54-0.45) | -2.221 | 0.026 | -0.209 |
| IQR - interquartile range, HADS-D - German Hospital Anxiety and Depression Scale, SSD-12 - Somatic Symptom Disorder - B Criteria Scale, ITQ - International Trauma Questionnaire, BFI - Brief Fatigue Inventory, FIS - Fatigue Impact Scale, MoCA - Montreal Cognitive Assessment, DSST - Digit Symbol Substitution Test (DSST_1: number of correct symbols within 90 sec., DSST_2: number of correct symbols from memory), TMT - Trail Making Test. | | | | | | | | | | | |

| **Table A7:** Groupwise comparison of psychological parameters, fatigue, and cognitive parameters of patients with medium and high socioeconomic status between timepoints T1 and T3. | | | | | | | | | | | |
| --- | --- | --- | --- | --- | --- | --- | --- | --- | --- | --- | --- |
|  | **Socioeconomic status** | | | | | | | | **Between-group** | | |
|  | **Medium** | | | | **High** | | | | **difference** | | |
|  | **N** | **T1**  **Median**  **(IQR)** | **T3**  **Median**  **(IQR)** | **Δ** | **N** | **T1**  **Median**  **(IQR)** | **T3**  **Median**  **(IQR)** | **Δ** | **z** | **p** | **r** |
| HADS-D_Depression_ | 40 | 8.00 (4.00 – 10.75) | 6.50 (3.00 – 9.00) | -1.00 (-2.00-1.00) | 73 | 7.00 (4.00 – 11.00) | 7.00 (3.00 – 11.00) | 0.00 (-3.00-2.00) | 0.646 | 0.519 | 0.061 |
| HADS-D_Anxiety_ | 40 | 7.00 (4.00 – 11.75) | 6.00 (4.00 – 9.75) | -0.50 (-3.00-1.00) | 73 | 6.00 (4.00 – 10.50) | 6.00 (3.00 – 9.50) | -1.00 (-3.00-2.00) | 0.576 | 0.564 | 0.054 |
| SSD-12 | 41 | 23.00 (18.50 – 30.50) | 24.00 (18.50 – 32.50) | 0.00 (-3.00-7.00) | 76 | 26.00 (17.00 – 33.00) | 23.00 (15.50 – 33.75) | -0.50 (-8.50-2.75) | -1.035 | 0.300 | -0.096 |
| ITQ | 41 | 7.00 (2.00 – 15.50) | 5.00 (1.50 – 10.00) | -2.00 (-4.00-1.00) | 75 | 8.00 (3.00 – 13.00) | 6.00 (1.00 – 12.00) | -1.00 (-4.00-2.00) | 0.683 | 0.495 | 0.063 |
| BFI | 41 | 5.44 (4.00 – 6.83) | 6.00 (5.28 – 6.78) | 0.33 (-0.61-1.33) | 76 | 5.61 (4.69 – 6.67) | 5.78 (4.53 – 6.97) | -0.11 (-0.88-1.06) | -1.277 | 0.201 | -0.118 |
| FIS | 40 | 98.50 (66.00 – 110.75) | 96.00 (64.00 – 114.50) | 1.50 (-5.25-15.75) | 76 | 95.50 (77.25 – 115.50) | 93.00 (72.25 – 113.00) | -2.00 (-20.00-11.75) | -1.612 | 0.107 | -0.150 |
| MoCA | 36 | 26.00 (25.00-28.00) | 26.50 (25.00-28.00) | 0.00 (-1.75-1.75) | 71 | 27.00 (26.00-29.00) | 27.00 (25.00-28.00) | 0.00 (-2.00-1.00) | -0.853 | 0.394 | -0.083 |
| DSST_1 | 36 | 42.00 (36.00-50.75) | 48.50 (38.50-56.00) | 2.00 (0.00-8.00) | 70 | 47.00 (38.50-54.25) | 52.00 (45.00-59.25) | 5.00 (-1.00-9.00) | -0.127 | 0.899 | -0.012 |
| DSST_2 | 36 | 4.00 (3.00-7.00) | 5.00 83.25-7.75) | 0.00 (-1.00-2.00) | 70 | 5.00 (2.00-6.00) | 7.00 (4.00-8.00) | 2.00 (0.00-3.00) | 1.854 | 0.064 | 0.180 |
| TMT-A | 36 | 34.76 (27.19 – 45.26) | 30.90 (24.85 – 38.95) | -3.45 (-9.60-4.74) | 71 | 33.00 (27.00 – 44.00) | 30.80 (23.00 – 38.00) | -4.36 (-1.18-3.00) | -0.204 | 0.838 | -0.020 |
| TMT-B | 35 | 72.00 (58.00 – 84.00) | 73.00 (60.27 – 88.00) | 0.68 (-17.00-10.78) | 70 | 72.08 (59.33 – 90.05) | 67.45 (47.93 – 82.65) | -7.86(-18.34-3.11) | -1.652 | 0.099 | -0.161 |
| Subj. mental health | 41 | 5.27 (4.45 – 6.77) | 5.09 (4.27 – 6.50) | -0.09 (-1.04-0.63) | 76 | 5.27 (4.25 – 6.27) | 5.09 (4.30 – 6.70) | 0.00 (-0.72-0.72) | 0.600 | 0.548 | 0.056 |
| IQR - interquartile range, HADS-D - German Hospital Anxiety and Depression Scale, SSD-12 - Somatic Symptom Disorder - B Criteria Scale, ITQ - International Trauma Questionnaire, BFI - Brief Fatigue Inventory, FIS - Fatigue Impact Scale, MoCA - Montreal Cognitive Assessment, DSST - Digit Symbol Substitution Test (DSST_1: number of correct symbols within 90 sec., DSST_2: number of correct symbols from memory), TMT - Trail Making Test. | | | | | | | | | | | |

| **Table A8:** Groupwise comparison of psychological parameters, fatigue, and cognitive parameters of patients with medium and high socioeconomic status between timepoints T1 and T4. | | | | | | | | | | | |
| --- | --- | --- | --- | --- | --- | --- | --- | --- | --- | --- | --- |
|  | **Socioeconomic status** | | | | | | | | **Between-group** | | |
|  | **medium SES** | | | | **high SES** | | | | **difference** | | |
|  | **N** | **T1**  **Median**  **(IQR)** | **T4**  **Median**  **(IQR)** | **Δ** | **N** | **T1**  **Median**  **(IQR)** | **T4**  **Median**  **(IQR)** | **Δ** | **z** | **p** | **r** |
| HADS-D_Depression_ | 35 | 7.00 (4.00 – 9.00) | 7.00 (4.00 – 11.00) | 1.00 (-2.00-2.00) | 69 | 7.00 (4.00 – 11.00) | 6.00 (3.50 – 11.00) | 0.00 (-2.50-2.00) | -0.518 | 0.604 | -0.051 |
| HADS-D_Anxiety_ | 35 | 6.00 (3.00 – 11.00) | 6.00 (3.00 – 11.00) | -1.00 (-3.00-2.00) | 69 | 7.00 (4.00 – 10.50) | 5.00 (3.00 – 9.00) | -1.00 (-3.00-1.00) | -0.411 | 0.681 | -0.040 |
| SSD-12 | 37 | 23.00 (18.50 – 28.00) | 23.00 (16.50 – 31.50) | 1.00 (-5.00-5.50) | 74 | 25.00 (16.75 – 32.25) | 22.50 (16.00 – 31.00) | -3.00 (-8.00-5.00) | -1.459 | 0.145 | -0.139 |
| ITQ | 37 | 6.00 (2.00 – 14.00) | 3.00 (0.50 – 10.00) | -1.00 (-6.00-1.00) | 74 | 8.00 (3.00 – 13.00) | 5.00 (1.00 – 9.00) | -2.00 (-5.00-0.00) | -0.694 | 0.488 | -0.066 |
| BFI | 37 | 5.11 (3.94 – 6.44) | 5.89 (4.78 – 7.06) | 0.88 (0.00-1.27) | 76 | 5.61 (4.72 – 6.67) | 6.06 (4.58 – 7.00) | 0.27 (-0.88-1.08) | -2.137 | 0.033 | -0.201 |
| FIS | 37 | 97.00 (64.50 – 110.00) | 93.00 (61.50 – 116.00) | 4.00 (-11.50-19.50) | 74 | 95.50 (76.50 – 114.50) | 100.50 (74.75 – 118.00) | -1.00 (-12.25-14.00) | -0.842 | 0.400 | -0.080 |
| MoCA | 33 | 26.00 (25.00-27.50) | 27.00 (25.50-28.00) | 1.00 (-1.00-1.00) | 64 | 27.00 (26.00-29.009 | 28.00 (26.00-29.00) | 0.00 (-1.00-2.00) | -0.736 | 0.462 | -0.075 |
| DSST_1 | 33 | 40.00 (34.50-50.00) | 47.00 (36.50-54.00) | 3.00 (-1.50-10.50) | 64 | 48.50 (40.25-54.75) | 51.00 (41.00-61.50) | 3.00 (-3.00-9.00) | -0.286 | 0.775 | -0.029 |
| DSST_2 | 33 | 4.00 (3.00-6.50) | 6.00 (4.00-7.00) | 1.00 (-0.50-2.00) | 64 | 5.00 (2.00-6.75) | 6.00 (4.00-8.00) | 2.00 (0.00-2.00) | 1.007 | 0.314 | 0.102 |
| TMT-A | 33 | 35.00 (27.88 – 47.50) | 32.36 (25.70 – 47.83) | -4.23 (-8.75-2.68) | 64 | 32.59 (26.36 – 40.77) | 27.47 (22.91 – 37.88) | -4.64 (-10.38-0.99) | -0.343 | 0.732 | -0.035 |
| TMT-B | 33 | 74.73 (59.15 – 100.59) | 73.00 (59.05 – 98.60) | -3.53 (-23.10-14.50) | 64 | 70.34 (57.22 – 84.75) | 61.10 (53.67 – 73.76) | -7.83 (-17.35-5.94) | -0.613 | 0.540 | -0.062 |
| Subj. mental health | 37 | 5.55 (4.64 – 6.91) | 5.18 (4.18 – 6.32) | -0.27 (-1.50-1.22) | 75 | 5.36 (4.45 – 6.36) | 5.27 (4.45 – 6.64) | 0.18 (-0.81-1.00) | 1.176 | 0.240 | 0.111 |
| IQR - interquartile range, HADS-D - German Hospital Anxiety and Depression Scale, SSD-12 - Somatic Symptom Disorder - B Criteria Scale, ITQ - International Trauma Questionnaire, BFI - Brief Fatigue Inventory, FIS - Fatigue Impact Scale, MoCA - Montreal Cognitive Assessment, DSST - Digit Symbol Substitution Test (DSST_1: number of correct symbols within 90 sec., DSST_2: number of correct symbols from memory), TMT - Trail Making Test. | | | | | | | | | | | |

| **Table A9:** Groupwise comparison of psychological parameters, fatigue, and cognitive parameters of healthcare workers and non-healthcare workers between timepoints T1 and T3. | | | | | | | | | | | |
| --- | --- | --- | --- | --- | --- | --- | --- | --- | --- | --- | --- |
|  | **Healthcare workers** | | | | **Non-healthcare workers** | | | | **Between-group**  **difference** | | |
|  | **N** | **T1**  **Median**  **(IQR)** | **T3**  **Median**  **(IQR)** | **Δ** | **N** | **T1**  **Median**  **(IQR)** | **T3**  **Median**  **(IQR)** | **Δ** | **z** | **p** | **r** |
| HADS-D_Depression_ | 79 | 8.00 (5.00 – 11.00) | 6.00 (3.00 – 10.00) | -1.00 (-3.00-1.00) | 35 | 6.00 (3.00 – 10.00) | 7.00 (3.00 – 13.00) | 0.00 (-1.00-3.00) | 2.321 | 0.020 | 0.217 |
| HADS-D_Anxiety_ | 79 | 7.00 (4.00 – 11.00) | 6.00 (3.00 – 10.00) | -1.00 (-3.00-1.00) | 35 | 5.00 (3.00 – 10.00) | 6.00 (3.00 – 8.00) | 0.00 (-2.00-2.00) | 1.195 | 0.232 | 0.112 |
| SSD-12 | 83 | 24.00 (17.00 – 33.00) | 23.00 (17.00 – 33.00) | -1.00 (-7.00-2.00) | 35 | 26.00 (21.00 – 31.00) | 24.00 (20.00 – 35.00) | 1.00 (-9.00-8.00) | 1.171 | 0.242 | 0.108 |
| ITQ | 83 | 8.00 (3.00 – 14.00) | 7.00 (2.00 – 11.00) | -1.00 (-5.00-2.00) | 34 | 6.00 (2.75 – 11.25) | 5.00 (1.00 – 10.00) | -1.00 (-3.00-1.00) | 0.196 | 0.845 | 0.018 |
| BFI | 83 | 5.56 (4.22 – 7.00) | 5.78 (4.89 – 6.78) | 0.11 (-0.88-1.00) | 35 | 5.44 (4.33 – 6.67) | 5.89 (5.22 – 7.00) | 0.33 (-0.66-1.77) | 1.031 | 0.302 | 0.095 |
| FIS | 83 | 98.00 (74.00 – 114.00) | 92.00 (67.00 – 113.00) | -1.00 (-20.00-12.00) | 34 | 93.50 (63.75 – 113.00) | 97.00 (70.75 – 115.75) | 7.00 (-6.25-14.25) | 1.901 | 0.057 | 0.176 |
| MoCA | 75 | 27.00 (25.00-28.00) | 27.00 (25.00-28.00) | 0.00 (-2.00-1.00) | 33 | 26.00 (25.00-28.00) | 27.00 (25.00-28.00) | 0.00 (-2.00-2.00) | 0.974 | 0.330 | 0.094 |
| DSST_1 | 75 | 47.00 (40.00-54.00) | 51.00 (45.00-60.00) | 5.00 (0.00-9.00) | 32 | 41.00 (34.00-50.75) | 48.00 (34.25-53.75) | 1.50 (-3.50-7.50) | -1.697 | 0.090 | -0.164 |
| DSST_2 | 75 | 4.00 (3.00-6.00) | 6.00 (4.00-8.00) | 2.00 (0.00-3.00) | 32 | 5.00 (2.25-6.00) | 5.00 (4.00-7.75) | -0.50 (-1.00-2.00) | -1.667 | 0.095 | -0.161 |
| TMT-A | 75 | 35.00 (28.00 – 44.00) | 29.00 (24.00 – 26.88) | -4.84 (-11.18-1.84) | 33 | 32.20 (25.97 – 43.43) | 33.89 (24.82 – 41.67) | 3.00 (-10.70-9.34) | 2.111 | 0.035 | 0.203 |
| TMT-B | 74 | 70.81 (57.58 – 88.02) | 67.00 (50.78 – 78.80) | -6.50 (-17.18-4.10) | 32 | 73.96 (61.97 – 99.75) | 73.20 (60.35 – 84.38) | -6.89 (-15.68-16.02) | 0.716 | 0.474 | 0.070 |
| Subj. mental health | 83 | 5.18 (4.09 – 6.27) | 5.09 (4.36 – 6.64) | 0.09 (-0.72-0.81) | 35 | 5.55 (4.73 – 7.00) | 5.00 (4.27 – 6.64) | -0.09 (-1.27-0.45) | -1.491 | 0.136 | -0.137 |
| IQR - interquartile range, HADS-D - German Hospital Anxiety and Depression Scale, SSD-12 - Somatic Symptom Disorder - B Criteria Scale, ITQ - International Trauma Questionnaire, BFI - Brief Fatigue Inventory, FIS - Fatigue Impact Scale, MoCA - Montreal Cognitive Assessment, DSST - Digit Symbol Substitution Test (DSST_1: number of correct symbols within 90 sec., DSST_2: number of correct symbols from memory), TMT - Trail Making Test. | | | | | | | | | | | |

| **Table A10:** Groupwise comparison of psychological parameters, fatigue, and cognitive parameters of healthcare workers and non-healthcare workers between timepoints T1 and T4. | | | | | | | | | | | |
| --- | --- | --- | --- | --- | --- | --- | --- | --- | --- | --- | --- |
|  | **Healthcare workers** | | | | **Non-healthcare workers** | | | | **Between-group**  **difference** | | |
|  | **N** | **T1**  **Median**  **(IQR)** | **T4**  **Median**  **(IQR)** | **Δ** | **N** | **T1**  **Median**  **(IQR)** | **T4**  **Median**  **(IQR)** | **Δ** | **z** | **p** | **r** |
| HADS-D_Depression_ | 75 | 7.00 (5.00 – 11.00) | 6.00 (4.00 – 11.00) | 0.00 (-3.00-2.00) | 30 | 5.50 (3.00 – 9.50) | 7.00 (2.75 – 12.25) | 0.50 (-2.00-2.25) | 1.407 | 0.159 | 0.137 |
| HADS-D_Anxiety_ | 75 | 7.00 (4.00 – 11.00) | 5.00 (3.00 – 10.00) | -1.00 (-4.00-1.00) | 30 | 5.00 (3.00 – 10.00) | 6.00 (2.75 – 9.25) | -0.50 (-2.25-2.00) | 1.407 | 0.159 | 0.137 |
| SSD-12 | 80 | 24.50 (17.00 – 31.75) | 23.00 (16.25 – 31.00) | -2.50 (-7.00-5.00) | 32 | 25.00 (16.50 – 30.75) | 23.50 (16.50 – 30.00) | -0.50 (-8.00-8.00) | 0.461 | 0.645 | 0.044 |
| ITQ | 79 | 8.00 (2.00 – 14.00) | 4.00 (1.00 – 10.00) | -2.00 (-5.00-0.00) | 33 | 6.00 (3.00 – 11.00) | 3.00 (1.00 – 7.50) | -2.00 (-5.00-1.00) | 0.759 | 0.448 | 0.072 |
| BFI | 80 | 5.56 (4.22 – 6.64) | 5.89 (4.47 – 6.97) | 0.33 (-0.66-1.11) | 34 | 5.44 (4.28 – 6.58) | 6.22 (5.42 – 7.03) | 0.72 (0.11-1.25) | 1.459 | 0.144 | 0.137 |
| FIS | 78 | 97.50 (74.00 – 113.25) | 97.00 (72.75 – 116.25) | 0.50 (-12.25-14.00) | 34 | 93.50 (63.75 – 109.00) | 96.50 (72.25 – 118.50) | 0.50 (-10.75-22.25) | 0.972 | 0.331 | 0.092 |
| MoCA | 71 | 27.00 826.00-28.00) | 28.00 (26.00-29.00) | 0.00 (-1.00-1.00) | 27 | 26.00 (25.00-27.009 | 27.00 (26.00-28.00) | 0.00 (-1.00-2.00) | 0.503 | 0.615 | 0.051 |
| DSST_1 | 71 | 47.00 (40.00-54.00) | 50.00 (41.00-60.00) | 3.00 (-3.00-10.00) | 27 | 41.00 (34.00-50.00) | 46.00 (35.00-54.00) | 4.00 (-3.00-8.00) | -0.032 | 0.975 | -0.003 |
| DSST_2 | 71 | 5.00 (2.00-7.00) | 6.00 (4.00-8.00) | 2.00 (0.00-2.00) | 27 | 5.00 (3.00-6.00) | 4.00 (3.00-7.00) | 1.00 (0.00-2.00) | -0.951 | 0.342 | -0.096 |
| TMT-A | 71 | 35.00 (27.00 – 47.00) | 28.60 (24.00 – 39.00) | -5.56 (-11.03-1.00) | 27 | 32.20 (25.93 – 41.00) | 29.48 (25.70 – 48.66) | 4.23 (-8.06-5.67) | 1.384 | 0.166 | 0.140 |
| TMT-B | 71 | 71.06 (57.77 – 89.00) | 64.73 (54.00 – 84.55) | -5.15 (-20.10-6.00) | 27 | 71.00 (58.23 – 85.00) | 69.00 (58.09 – 87.00) | -6.70 (-15.43-20.00) | 1.018 | 0.309 | 0.103 |
| Subj. mental health | 80 | 5.27 (4.18 – 6.34) | 5.14 (4.27 – 6.55) | 0.00 (-0.79-0.97) | 33 | 5.55 (4.91 – 7.09) | 5.91 (4.50 – 6.68) | 0.09 (-1.06-1.18) | -0.253 | 0.801 | -0.024 |
| IQR - interquartile range, HADS-D - German Hospital Anxiety and Depression Scale, SSD-12 - Somatic Symptom Disorder - B Criteria Scale, ITQ - International Trauma Questionnaire, BFI - Brief Fatigue Inventory, FIS - Fatigue Impact Scale, MoCA - Montreal Cognitive Assessment, DSST - Digit Symbol Substitution Test (DSST_1: number of correct symbols within 90 sec., DSST_2: number of correct symbols from memory), TMT - Trail Making Test. | | | | | | | | | | | |

| **Table A11:** Groupwise comparison of psychological parameters, fatigue, and cognitive parameters of patients with pre-existing cardiovascular disease and without a pre-existing cardiovascular disease between timepoints T1 and T3. | | | | | | | | | | | |
| --- | --- | --- | --- | --- | --- | --- | --- | --- | --- | --- | --- |
|  | **Pre-existing cardiovascular disease** | | | | | | | | **Between-group** | | |
|  | **No** | | | | **Yes** | | | | **difference** | | |
|  | **N** | **T1**  **Median**  **(IQR)** | **T3**  **Median**  **(IQR)** | **Δ** | **N** | **T1**  **Median**  **(IQR)** | **T3**  **Median**  **(IQR)** | **Δ** | **z** | **p** | **r** |
| HADS-D_Depression_ | 60 | 7.00 (4.00 – 9.00) | 6.00 (3.00 – 9.00) | -0.50 (-2.00-1.00) | 54 | 9.00 (4.00 – 12.00) | 8.00 (3.00 – 11.25) | -0.50 (-3.00-2.00) | 0.046 | 0.964 | 0.004 |
| HADS-D_Anxiety_ | 60 | 6.00 (3.00 – 10.00) | 6.00 (3.00 – 8.75) | 0.00 (-2.00-1.00) | 54 | 7.50 (4.00 – 11.00) | 6.50 (3.00 – 10.00) | -1.50 (-3.00-2.00) | -0.644 | 0.519 | -0.060 |
| SSD-12 | 62 | 25.00 (15.75 – 32.25) | 23.00 (16.25 – 32.25) | -1.00 (-7.00-6.00) | 56 | 24.50 (18.00 – 32.00) | 24.00 (18.00 – 33.75) | 0.50 (-6.50-3.00) | 0.378 | 0.706 | 0.035 |
| ITQ | 62 | 6.50 (2.00 – 14.00) | 5.00 (1.00 – 10.00) | -1.00 (-4.25-2.00) | 55 | 8.00 (4.00 – 14.00) | 6.00 (2.00 – 12.00) | -2.00 (-4.00-1.00) | -0.298 | 0.765 | -0.028 |
| BFI | 62 | 5.47 (4.31 – 6.47) | 5.78 (4.75 – 6.78) | 0.13 (-0.69-1.22) | 56 | 5.72 (4.28 – 7.08) | 5.89 (5.03 – 6.97) | 0.16 (-0.86-1.05) | -0.094 | 0.925 | -0.009 |
| FIS | 61 | 88.00 (73.50 – 112.00) | 91.00 (71.50 – 112.00) | 0.00 (-11.00-14.00) | 56 | 99.00 (71.25 – 116.25) | 97.00 (65.50 – 115.00) | -1.00 (-11.50-11.75) | -0.379 | 0.704 | -0.035 |
| MoCA | 58 | 27.00 (26.00-29.00) | 27.00 (25.75-29.00) | 0.00 (-2.00-1.00) | 50 | 26.00 (24.75-27.00) | 26.00 (24.00-27.00) | 0.00 (-3.00-1.00) | -0.311 | 0.756 | -0.030 |
| DSST_1 | 57 | 50.00 840.00-55.50) | 53.00 (45.00-60.00) | 3.00 (-2.00-9.00) | 50 | 42.00 (34.75-50.00) | 49.50 (38.00-53.25) | 5.00 (-0.25-8.25) | 0.650 | 0.516 | 0.063 |
| DSST_2 | 57 | 6.00 (3.50-7.00) | 7.00 (5.00-9.00) | 1.00 (0.00-2.00) | 50 | 4.00 (2.00-5.00) | 5.00 (3.00-7.00) | 1.50 (-1.00-3.25) | 0.353 | 0.724 | 0.034 |
| TMT-A | 58 | 32.04 (26.93 – 41.00) | 28.60 (23.00 – 36.67) | -4.40 (-12.09-3.31) | 50 | 34.85 (27.57 – 47.00) | 31.20 (25.76 – 39.35) | -4.18 (-10.29-4.04) | 0.185 | 0.853 | 0.018 |
| TMT-B | 56 | 71.08 (57.83 – 88.77) | 69.59 (47.18 – 79.90) | -5.54 (-19.37-4.68) | 50 | 73.41 (59.33 – 95.25) | 69.30 (53.98 – 85.63) | -8.41 (-16.18-7.47) | 0.307 | 0.759 | 0.030 |
| Subj. mental health | 62 | 5.36 (4.55 – 6.30) | 5.23 (4.64 – 6.68) | 0.18 (-1.00-0.72) | 56 | 5.04 (3.81 – 6.64) | 5.00 (4.11 – 6.59) | -0.04 (-0.63-0.52) | -0.108 | 0.914 | -0.010 |
| IQR - interquartile range, HADS-D - German Hospital Anxiety and Depression Scale, SSD-12 - Somatic Symptom Disorder - B Criteria Scale, ITQ - International Trauma Questionnaire, BFI - Brief Fatigue Inventory, FIS - Fatigue Impact Scale, MoCA - Montreal Cognitive Assessment, DSST - Digit Symbol Substitution Test (DSST_1: number of correct symbols within 90 sec., DSST_2: number of correct symbols from memory), TMT - Trail Making Test. | | | | | | | | | | | |

| **Table A12:** Groupwise comparison of psychological parameters, fatigue, and cognitive parameters of patients with pre-existing cardiovascular disease and without a pre-existing cardiovascular disease between timepoints T1 and T4. | | | | | | | | | | | |
| --- | --- | --- | --- | --- | --- | --- | --- | --- | --- | --- | --- |
|  | **Pre-existing cardiovascular disease** | | | | | | | | **Between-group** | | |
|  | **No** | | | | **Yes** | | | | **difference** | | |
|  | **N** | **T1**  **Median**  **(IQR)** | **T4**  **Median**  **(IQR)** | **Δ** | **N** | **T1**  **Median**  **(IQR)** | **T4**  **Median**  **(IQR)** | **Δ** | **z** | **p** | **r** |
| HADS-D_Depression_ | 54 | 6.00 (3.75 – 9.00) | 5.50 (3.75 – 10.00) | 0.00 (-3.00-2.00) | 51 | 8.00 (4.00 – 12.00) | 7.00 (4.00 – 12.00) | 0.00 (-2.00-2.00) | -0.006 | 0.995 | -0.001 |
| HADS-D_Anxiety_ | 54 | 6.00 (3.00 – 9.25) | 5.00 (3.00 – 9.25) | 0.00 (-3.00-1.25) | 51 | 7.00 (4.00 – 11.00) | 6.00 (3.00 – 10.00) | -1.00 (-4.00-2.00) | -1.050 | 0.294 | -0.103 |
| SSD-12 | 60 | 25.00 (15.25 – 32.75) | 23.00 (18.00 – 31.75) | -2.00 (-7.00-6.75) | 52 | 23.50 (17.25 – 29.50) | 23.50 (13.75 – 30.00) | -1.50 (-8.00-4.00) | -0.347 | 0.728 | -0.033 |
| ITQ | 59 | 7.00 (2.00 – 14.00) | 3.00 (1.00 – 10.00) | -2.00 (-5.00-0.00) | 53 | 7.00 (3.50 – 12.50) | 4.00 (1.00 – 8.50) | -2.00 (-5.50-0.50) | -0.026 | 0.979 | -0.003 |
| BFI | 60 | 5.47 (4.25 – 6.44) | 5.94 (5.03 – 6.78) | 0.55 (-0.33-1.22) | 54 | 5.50 (4.08 – 6.75) | 6.06 (4.19 – 7.00) | 0.27 (-0.71-1.11) | -1.346 | 0.178 | -0.126 |
| FIS | 59 | 88.00 (74.00 – 111.00) | 95.00 (74.00 – 117.00) | 1.00 (-13.00-15.00) | 53 | 99.00 (70.00 – 113.00) | 98.00 (71.00 – 117.50) | 0.00 (-11.50-14.00) | 0.029 | 0.977 | 0.003 |
| MoCA | 51 | 27.00 (26.00-29.00) | 28.00 (26.00-29.00) | 0.00 (-2.00-1.00) | 47 | 26.00 (24.00-27.00) | 27.00 (25.00-28.00) | 0.00 (-1.00-2.00) | 0.342 | 0.733 | 0.035 |
| DSST_1 | 51 | 50.00 (40.00-55.00) | 50.00 (43.00-63.00) | 2.00 (-4.00-9.00) | 47 | 42.00 (35.00-50.00) | 46.00 (36.00-56.00) | 5.00 (-1.00-10.00) | 1.117 | 0.264 | 0.113 |
| DSST_2 | 51 | 6.00 (3.00-7.00) | 7.00 (5.00-9.00) | 1.00 (0.00-2.00) | 47 | 4.00 (2.00-5.00) | 6.00 (4.00-7.00) | 2.00 (0.00-2.00) | 0.353 | 0.724 | 0.034 |
| TMT-A | 51 | 32.00 (26.71 – 41.00) | 27.00 (22.00 – 38.10) | -5.14 (-14.00-1.54) | 47 | 35.00 (27.76 – 47.00) | 30.00 (25.40 – 42.23) | -4.23 (-8.06-2.00) | 0.736 | 0.462 | 0.074 |
| TMT-B | 51 | 71.00 (56.00 – 89.00) | 64.00 (55.70 – 73.48) | -8.39 (-22.20-4.30) | 47 | 71.06 (58.23 – 84.00) | 70.10 (54.70 – 101.00) | 0.46 (-13.40-16.08) | 2.226 | 0.026 | 0.225 |
| Subj. mental health | 60 | 5.36 (4.57 – 6.27) | 5.23 (4.45 – 6.64) | 0.00 (-0.72-0.88) | 53 | 5.45 (4.00 – 6.86) | 5.27 (4.18 – 6.59) | 0.09 (-1.22-1.09) | -0.311 | 0.756 | -0.029 |
| IQR - interquartile range, HADS-D - German Hospital Anxiety and Depression Scale, SSD-12 - Somatic Symptom Disorder - B Criteria Scale, ITQ - International Trauma Questionnaire, BFI - Brief Fatigue Inventory, FIS - Fatigue Impact Scale, MoCA - Montreal Cognitive Assessment, DSST - Digit Symbol Substitution Test (DSST_1: number of correct symbols within 90 sec., DSST_2: number of correct symbols from memory), TMT - Trail Making Test. | | | | | | | | | | | |

| **Table A13:** Groupwise comparison of psychological parameters, fatigue, and cognitive parameters of patients with pre-existing respiratory disease and without a pre-existing respiratory disease between timepoints T1 and T3. | | | | | | | | | | | |
| --- | --- | --- | --- | --- | --- | --- | --- | --- | --- | --- | --- |
|  | **Pre-existing respiratory disease** | | | | | | | | **Between-group** | | |
|  | **No** | | | | **Yes** | | | | **difference** | | |
|  | **N** | **T1**  **Median**  **(IQR)** | **T3**  **Median**  **(IQR)** | **Δ** | **N** | **T1**  **Median**  **(IQR)** | **T3**  **Median**  **(IQR)** | **Δ** | **z** | **p** | **r** |
| HADS-D_Depression_ | 64 | 8.00 (5.00 – 11.00) | 6.00 (3.25 – 10.00) | 0.00 (-3.00-1.75) | 50 | 7.00 (3.00 – 11.25) | 7.00 (3.00 – 11.00) | -1.00 (-2.00-2.00) | 0.382 | 0.703 | 0.036 |
| HADS-D_Anxiety_ | 64 | 7.00 (3.25 – 11.00) | 6.00 (3.00 – 8.75) | -1.00 (-3.00-1.00) | 50 | 5.00 (4.00 – 10.00) | 7.00 (2.00 – 10.00) | 0.00 (-3.00-3.00) | 1.154 | 0.249 | 0.108 |
| SSD-12 | 66 | 24.50 (16.00 – 30.00) | 23.50 (16.50 – 32.00) | 0.00 (-5.50-4.50) | 52 | 26.00 (21.00 – 34.00) | 23.50 (19.00 – 33.75) | 0.00 (-8.50-3.00) | -0.396 | 0.692 | -0.037 |
| ITQ | 65 | 7.00 (2.00 – 14.00) | 5.00 (1.00 – 10.00) | -2.00 (-4.00-1.50) | 52 | 7.50 (3.00 – 13.00) | 6.50 (1.25 – 11.75) | -1.00 (-4.00-2.00) | 0.707 | 0.480 | 0.065 |
| BFI | 66 | 5.44 (4.31 – 6.78) | 5.89 (4.83 – 6.92) | 0.16 (-0.69-1.25) | 52 | 5.56 (4.24 – 6.67) | 5.83 (4.92 – 6.64) | 0.13 (-0.86-0.88) | -0.835 | 0.404 | -0.077 |
| FIS | 66 | 95.00 (72.50 – 111.50) | 94.50 (65.75 – 113.25) | 0.00 (-8.00-15.00) | 51 | 99.00 (72.00 – 116.00) | 92.00 (71.00 – 113.00) | -1.00 (-17.00-12.00) | -0.987 | 0.324 | -0.091 |
| MoCA | 61 | 27.00 (25.00-28.00) | 26.00 (24.50-28.00) | 0.00 (-2.00-1.00) | 47 | 27.00 (26.00-28.00) | 27.00 (25.00-28.00) | -0.00 (-2.00-1.00) | 0.097 | 0.923 | 0.009 |
| DSST_1 | 60 | 46.00 (37.00-52.00) | 50.00 (44.25-57.75) | 2.50 (-1.00-9.00) | 47 | 45.00 (37.00-54.00) | 50.00 (44.00-57.00) | 5.00 (-1.00-8.00) | -0.160 | 0.873 | -0.016 |
| DSST_2 | 60 | 4.00 (2.25-6.00) | 6.00 (4.00-8.00) | 1.50 (0.00-3.00) | 47 | 5.00 (3.00-6.00) | 7.00 (4.00-8.00) | 1.00 (-1.00-3.00) | -0.481 | 0.630 | -0.047 |
| TMT-A | 61 | 34.83 (26.86 – 40.05) | 30.70 (24.90 – 38.47) | -3.00 (-10.31-3.00) | 47 | 32.20 (27.00 – 45.86) | 31.00 (23.00 – 39.40) | -4.98 (-11.90-5.94) | -0.105 | 0.916 | -0.010 |
| TMT-B | 60 | 73.86 (61.50 – 91.75) | 70.64 (53.93 – 87.25) | -6.70 (-23.10-8.65) | 46 | 69.73 (55.95 – 88.02) | 62.71 (50.95 – 81.78) | 6.04 (-12.54-4.42) | 0.022 | 0.982 | 0.002 |
| Subj. mental health | 66 | 5.32 (4.45 – 6.36) | 5.27 (4.36 – 6.64) | 0.22 (-0.72-0.75) | 52 | 5.18 (4.27 – 6.68) | 4.95 (4.00 – 6.57) | -0.13 (-0.97-0.52) | -1.269 | 0.204 | -0.117 |
| IQR - interquartile range, HADS-D - German Hospital Anxiety and Depression Scale, SSD-12 - Somatic Symptom Disorder - B Criteria Scale, ITQ - International Trauma Questionnaire, BFI - Brief Fatigue Inventory, FIS - Fatigue Impact Scale, MoCA - Montreal Cognitive Assessment, DSST - Digit Symbol Substitution Test (DSST_1: number of correct symbols within 90 sec., DSST_2: number of correct symbols from memory), TMT - Trail Making Test. | | | | | | | | | | | |

| **Table A14:** Groupwise comparison of psychological parameters, fatigue, and cognitive parameters of patients with pre-existing respiratory disease and without a pre-existing respiratory disease between timepoints T1 and T4. | | | | | | | | | | | |
| --- | --- | --- | --- | --- | --- | --- | --- | --- | --- | --- | --- |
|  | **Pre-existing respiratory disease** | | | | | | | | **Between-group** | | |
|  | **No** | | | | **Yes** | | | | **difference** | | |
|  | **N** | **T1**  **Median**  **(IQR)** | **T4**  **Median**  **(IQR)** | **Δ** | **N** | **T1**  **Median**  **(IQR)** | **T4**  **Median**  **(IQR)** | **Δ** | **z** | **p** | **r** |
| HADS-D_Depression_ | 59 | 7.00 (5.00 – 10.00) | 7.00 (4.00 – 11.00) | 0.00 (-2.00-2.00) | 46 | 6.50 (3.00 – 11.25) | 6.00 (3.75 – 10.25) | -0.50 (-2.25-2.00) | -0.477 | 0.634 | -0.047 |
| HADS-D_Anxiety_ | 59 | 7.00 (3.00 – 11.00) | 6.00 (3.00 – 10.00) | -1.00 (-3.00-1.00) | 46 | 5.00 (4.00 – 10.25) | 5.00 (3.00 – 9.25) | -0.50 (-3.00-2.00) | 0.555 | 0.579 | 0.054 |
| SSD-12 | 64 | 25.00 (16.00 – 28.75) | 22.50 (15.25 – 31.00) | -1.00 (-6.75-6.00) | 48 | 24.50 (20.25 – 34.00) | 23.50 (19.00 – 30.75) | -3.00 (-8.75-4.00) | -1.142 | 0.254 | -0.108 |
| ITQ | 63 | 7.00 (2.00 – 14.00) | 5.00 (1.00 – 11.00) | -2.00 (-6.00-1.00) | 49 | 7.00 (3.00 – 12.00) | 3.00 (1.00 – 8.00) | -2.00 (-5.00-0.00) | -0.409 | 0.682 | -0.039 |
| BFI | 65 | 5.44 (4.28 – 6.67) | 6.11 (5.00 – 7.00) | 0.66 (-0.66-1.22) | 49 | 5.56 (4.11 – 6.56) | 5.67 (4.28 – 7.00) | 0.33 (-0.55-1.05) | -1.137 | 0.256 | -0.107 |
| FIS | 63 | 95.00 (73.00 – 113.00) | 98.00 (69.00 – 123.00) | 2.00 (-10.00-18.00) | 49 | 97.00 (72.00 – 111.50) | 95.00 (75.50 – 108.50) | -2.00 (-15.50-12.50) | -1.211 | 0.226 | -0.114 |
| MoCA | 56 | 27.00 825.00-28.00) | 27.00 (26.00-28.75) | 0.00 (-1.00-2.00) | 42 | 27.00 (26.00-29.00) | 27.00 (25.00-28.00) | 0.00 (-2.00-1.00) | -1.307 | 0.191 | -0.132 |
| DSST_1 | 56 | 46.00 (37.00-51.00) | 49.00 (38.25-56.00) | 3.00 (-3.00-10.00) | 42 | 46.00 (36.75-54.50) | 49.50 (36.75-60.75) | 4.00 (-1.25-8.00) | 0.018 | 0.986 | 0.002 |
| DSST_2 | 56 | 4.00 (2.00-6.75) | 6.00 (4.00-8.00) | 2.00 (0.00-3.00) | 42 | 5.00 (3.00-6.25) | 6.00 (4.00-8.00) | 1.00 (0.00-2.00) | -0.481 | 0.630 | -0.047 |
| TMT-A | 56 | 34.42 (26.78 – 44.42) | 29.53 (24.94 – 40.91) | -4.14 (-9.97-2.07) | 42 | 33.85 (27.57 – 44.47) | 28.70 (23.70 – 42.90) | -5.07 (-10.61-1.13) | 0.050 | 0.960 | 0.005 |
| TMT-B | 56 | 73.41 (61.00 – 90.72) | 67.25 (55.36 – 88.50) | -6.16 (-22.11-7.79) | 42 | 68.03 (55.54 – 88.02) | 63.13 (54.39 – 75.08) | -5.36 (-14.72-5.82) | 0.567 | 0.571 | 0.057 |
| Subj. mental health | 64 | 5.36 (4.48 – 6.36) | 5.18 (4.39 – 6.55) | -0.13 (-1.06-0.97) | 49 | 5.36 (4.55 – 6.82) | 5.55 (4.27 – 6.64) | 0.18 (-0,71-1.09) | 0.846 | 0.397 | 0.037 |
| IQR - interquartile range, HADS-D - German Hospital Anxiety and Depression Scale, SSD-12 - Somatic Symptom Disorder - B Criteria Scale, ITQ - International Trauma Questionnaire, BFI - Brief Fatigue Inventory, FIS - Fatigue Impact Scale, MoCA - Montreal Cognitive Assessment, DSST - Digit Symbol Substitution Test (DSST_1: number of correct symbols within 90 sec., DSST_2: number of correct symbols from memory), TMT - Trail Making Test. | | | | | | | | | | | |

| **Table A15:** Groupwise comparison of psychological parameters, fatigue, and cognitive parameters of patients with pre-existing metabolic disease and without a pre-existing metabolic disease between timepoints T1 and T3. | | | | | | | | | | | |
| --- | --- | --- | --- | --- | --- | --- | --- | --- | --- | --- | --- |
|  | **Pre-existing metabolic disease** | | | | | | | | **Between-group** | | |
|  | **No** | | | | **Yes** | | | | **difference** | | |
|  | **N** | **T1**  **Median**  **(IQR)** | **T3**  **Median**  **(IQR)** | **Δ** | **N** | **T1**  **Median**  **(IQR)** | **T3**  **Median**  **(IQR)** | **Δ** | **z** | **p** | **r** |
| HADS-D_Depression_ | 39 | 6.00 (3.00 – 9.00) | 6.00 (3.00 – 10.00) | 0.00 (-2.00-2.00) | 75 | 9.00 (4.00 – 12.00) | 7.00 (3.00 – 11.00) | -1.00 (-3.00-2.00) | -0.765 | 0.444 | -0.072 |
| HADS-D_Anxiety_ | 39 | 6.00 (3.00 – 9.00) | 5.00 (3.00 – 8.00) | 0.00 (-3.00-2.00) | 75 | 7.00 (4.00 – 11.00) | 7.00 (3.00 – 10.00) | -1.00 (-3.00-1.00) | -0.039 | 0.969 | -0.004 |
| SSD-12 | 40 | 25.00 (15.00 – 29.75) | 21.50 (12.00 – 30.75) | -0.50 (-8.50-2.00) | 78 | 25.00 (18.75 – 33.00) | 24.00 (19.00 – 33.25) | 0.00 (-5.50-4.75) | 0.959 | 0.337 | 0.088 |
| ITQ | 39 | 6.00 (1.00 – 11.00) | 5.00 (1.00 – 10.00) | -1.00 (-3.00-2.00) | 78 | 7.50 (3.00 – 14.00) | 6.00 (2.00 – 11.00) | -2.00 (-4.25-1.25) | -1.139 | 0.255 | -0.105 |
| BFI | 40 | 5.17 (4.22 – 6.11) | 5.78 (4.11 – 6.94) | 0.22 (-0.66-1.22) | 78 | 5.72 (4.75 – 7.03) | 5.89 (5.08 – 6.81) | 0.13 (-0.80-1.00) | -0.276 | 0.783 | -0.025 |
| FIS | 39 | 85.00 (64.00 – 111.00) | 88.00 (66.00 – 113.00) | 0.00 (-8.00-15.00) | 70 | 99.00 (74.00 – 117.00) | 96.50 (74.50 – 114.25) | 0.00 (-12.25-11.25) | -0.633 | 0.527 | -0.059 |
| MoCA | 38 | 27.00 (25.00-29.00) | 27.00 (24.75-29.00) | -1.00 (-2.00-1.00) | 69 | 27.00 (25.00-28.00) | 27.00 (25.00-28.00) | 0.00 (-1.25-1.00) | 1.261 | 0.207 | 0.121 |
| DSST_1 | 38 | 50.00 (41.75-56.00) | 53.00 (46.50-59.75) | 2.00 (-5.00-9.25) | 69 | 43.00 (35.00-52.00) | 49.00 (41.50-56.00) | 5.00 (0.00-9.00) | 0.952 | 0.341 | 0.092 |
| DSST_2 | 38 | 5.00 (3.00-6.25) | 7.00 (4.00-9.00) | 2.00 (0.00-3.25) | 69 | 4.00 (2.50-6.00) | 6.00 (4.00-8.00) | 1.00 (-1.00-3.00) | -1.195 | 0.232 | -0.116 |
| TMT-A | 38 | 32.00 (24.00 – 40.31) | 26.88 (21.04 – 35.85) | -4.58 (-12.22-2.43) | 70 | 35.00 (28.00 – 47.00) | 31.30 (26.75 – 39.10) | -3.78 (-9.89-5.71) | 0.589 | 0.556 | 0.057 |
| TMT-B | 38 | 68.81 (55.95 – 90.16) | 62.50 (46.30 – 79.70) | -7.60 (-21.44-1.78) | 68 | 74.00 (64.22 – 88.77) | 72.14 (55.00 – 84.13) | -5.76 (-16.82-6.94) | 1.024 | 0.306 | 0.100 |
| Subj. mental health | 40 | 5.50 (4.77 – 6.36) | 5.50 (4.75 – 6.64) | 0.00 (-0.72-0.79) | 78 | 5.09 (3.98 – 6.45) | 5.00 (4.11 – 6.64) | 0.00 (-0.77-0.63) | -0.384 | 0.701 | -0.035 |
| IQR - interquartile range, HADS-D - German Hospital Anxiety and Depression Scale, SSD-12 - Somatic Symptom Disorder - B Criteria Scale, ITQ - International Trauma Questionnaire, BFI - Brief Fatigue Inventory, FIS - Fatigue Impact Scale, MoCA - Montreal Cognitive Assessment, DSST - Digit Symbol Substitution Test (DSST_1: number of correct symbols within 90 sec., DSST_2: number of correct symbols from memory), TMT - Trail Making Test. | | | | | | | | | | | |

| **Table A16:** Groupwise comparison of psychological parameters, fatigue, and cognitive parameters of patients with pre-existing metabolic disease and without a pre-existing metabolic disease between timepoints T1 and T4. | | | | | | | | | | | |
| --- | --- | --- | --- | --- | --- | --- | --- | --- | --- | --- | --- |
|  | **Pre-existing metabolic disease** | | | | | | | | **Between-group** | | |
|  | **No** | | | | **Yes** | | | | **difference** | | |
|  | **N** | **T1**  **Median**  **(IQR)** | **T4**  **Median**  **(IQR)** | **Δ** | **N** | **T1**  **Median**  **(IQR)** | **T4**  **Median**  **(IQR)** | **Δ** | **z** | **p** | **r** |
| HADS-D_Depression_ | 39 | 5.00 (3.00 – 9.00) | 6.00 (3.00 – 10.00) | 1.00 (-1.00-2.00) | 66 | 7.50 (4.00 – 12.00) | 6.50 (4.00 – 11.25) | -1.00 (-3.00-2.00) | -1.515 | 0.130 | -0.148 |
| HADS-D_Anxiety_ | 39 | 6.00 (3.00 – 8.00) | 5.00 (3.00 – 9.00) | -1.00 (-2.00-1.00) | 66 | 7.00 (4.00 – 11.00) | 6.00 (4.00 – 10.00) | -1.00 (-3.00-2.00) | -0.290 | 0.772 | -0.028 |
| SSD-12 | 41 | 25.00 (15.00 – 29.50) | 23.00 (10.00 – 30.50) | -1.00 (-5.00-5.00) | 66 | 24.00 (18.00 – 33.00) | 23.00 (18.00 – 31.00) | -2.00 (-8.00-6.00) | -0.242 | 0.809 | -0.023 |
| ITQ | 41 | 6.00 (1.00 – 11.00) | 2.00 (0.00 – 7.50) | -1.00 (-5.00-1.00) | 66 | 7.00 (4.00 – 14.00) | 5.00 (1.00 – 10.00) | -2.00 (-5.00-0.00) | -0.749 | 0.454 | -0.071 |
| BFI | 42 | 5.22 (4.22 – 6.14) | 6.17 (4.17 – 6.83) | 0.38 (-0.66-1.22) | 71 | 5.56 (4.64 – 6.67) | 5.89 (5.00 – 7.00) | 0.41 (-0.63-1.19) | -0.182 | 0.855 | -0.017 |
| FIS | 41 | 87.00 (66.50 – 112.00) | 94.00 (60.00 – 118.00) | 0.00 (-12.50-13.50) | 71 | 99.00 (74.00 – 112.00) | 98.00 (76.00 – 116.00) | 1.00 (-12.00-17.00) | 0.432 | 0.666 | 0.041 |
| MoCA | 36 | 27.00 (25.00-29.009 | 27.50 (26.00-29.00) | 0.00 (-1.75-2.75) | 72 | 27.00 (25.75-28.00) | 27.00 (25.00-28.00) | 0.00 (-1.00-1.00) | -0.168 | 0.867 | -0.017 |
| DSST_1 | 36 | 50.00 (41.25-56.00) | 52.50 (45.25-65.00) | 4.00 (-3.75-10.00) | 71 | 42.50 (35.00-51.25) | 46.00 (36.00-56.25) | 2.00 (-2.25-7.25) | -0.564 | 0.573 | -0.057 |
| DSST_2 | 36 | 5.00 (3.00-6.75) | 7.00 (5.00-8.00) | 2.00 (0.00-3.00) | 62 | 4.00 (2.00-6.25) | 5.50 (3.75-7.00) | 1.00 (0.00-2.00) | -0.881 | 0.378 | -0.089 |
| TMT-A | 36 | 32.00 (24.00 – 40.77) | 27.00 (20.24 – 34.09) | -4.64 (-9.97- -0.51) | 62 | 35.46 (28.09 – 47.25) | 30.13 (24.98 – 45.50) | -4.89 (-10.60-2.14) | 0.188 | 0.851 | 0.019 |
| TMT-B | 36 | 67.00 (55.84 – 88.66) | 66.40 (55.26 – 83.91) | -7.22 (-19.82-6.15) | 62 | 73.86 (62.49 – 88.32) | 65.37 (54.61 – 87.50) | -5.07 (-20.25-8.20) | 0.088 | 0.930 | 0.009 |
| Subj. mental health | 41 | 5.55 (4.91 – 6.64) | 5.64 (4.86 – 6.86) | 0.00 (-1.00-1.09) | 72 | 5.18 (4.05 – 6.64) | 5.05 (4.20 – 6.50) | 0.04 (-0.79-1.00) | 0.266 | 0.790 | 0.025 |
| IQR - interquartile range, HADS-D - German Hospital Anxiety and Depression Scale, SSD-12 - Somatic Symptom Disorder - B Criteria Scale, ITQ - International Trauma Questionnaire, BFI - Brief Fatigue Inventory, FIS - Fatigue Impact Scale, MoCA - Montreal Cognitive Assessment, DSST - Digit Symbol Substitution Test (DSST_1: number of correct symbols within 90 sec., DSST_2: number of correct symbols from memory), TMT - Trail Making Test. | | | | | | | | | | | |

| **Table A17:** Groupwise comparison of psychological parameters, fatigue, and cognitive parameters of patients with pre-existing psychological disease and without a pre-existing psychological disease between timepoints T1 and T3. | | | | | | | | | | | |
| --- | --- | --- | --- | --- | --- | --- | --- | --- | --- | --- | --- |
|  | **Pre-existing psychological disease** | | | | | | | | **Between-group** | | |
|  | **No** | | | | **Yes** | | | | **difference** | | |
|  | **N** | **T1**  **Median**  **(IQR)** | **T3**  **Median**  **(IQR)** | **Δ** | **N** | **T1**  **Median**  **(IQR)** | **T3**  **Median**  **(IQR)** | **Δ** | **z** | **p** | **r** |
| HADS-D_Depression_ | 94 | 6.00 (3.00 – 10.00) | 6.00 (3.00 – 9.25) | 0.00 (-2.00-1.00) | 20 | 11.00 (8.00 – 13.00) | 11.00 (5.50 – 13.00) | -2.00 (-5.50-2.75) | -0.663 | 0.508 | -0.062 |
| HADS-D_Anxiety_ | 94 | 5.50 (3.00 – 10.00) | 6.00 (3.00 – 8.00) | 0.00 (-3.00-2.00) | 20 | 9.50 (8.00 – 13.75) | 9.50 (6.00 – 12.75) | -2.00 (-2.75-0.00) | -0.966 | 0.334 | -0,090 |
| SSD-12 | 96 | 23.50 (15.25 – 30.75) | 23.00 (15.00 – 32.00) | 0.00 (-7.00-5.50) | 22 | 30.00 (24.00 – 34.25) | 29.50 (20.50 – 35.00) | -0.50 (-5.50-3.00) | -0.360 | 0.719 | -0.033 |
| ITQ | 96 | 6.00 (2.00 – 11.00) | 4.50 (1.00 – 9.75) | -1.00 (-3.00-2.00) | 21 | 15.00 (10.00 – 19.50) | 11.00 (8.00 – 15.50) | -4.00 (-8.00-0.50) | -2.328 | 0.020 | -0.215 |
| BFI | 96 | 5.33 (4.14 – 6.53) | 5.78 (4.81 – 7.03) | 0.33 (-0.66-1.30) | 22 | 6.50 (5.39 – 7.67) | 6.17 (5.27 – 6.64) | -0.41 (-1.13-0.44) | -2.464 | 0.014 | -0.089 |
| FIS | 95 | 89.00 (71.00 – 110.00) | 92.00 (67.00 – 113.00) | 0.00 (-10.00-13.00) | 22 | 110.50 (90.00 – 120.75) | 99.50 (76.75 – 114.25) | -1.50 (-22.00-6.75) | -1.158 | 0.247 | -0.107 |
| MoCA | 89 | 27.00 (25.00-28.00) | 27.00 (25.00-28.00) | 0.00 (-2.00-1.00) | 19 | 26.00 (25.00-27.00) | 26.00 (24.00-27.00) | 0.00 (-2.00-1.00) | -0.090 | 0.929 | -0.009 |
| DSST_1 | 88 | 46.00 (37.25-53.00) | 51.00 (45.00-58.00) | 5.00 (-1.00-9.00) | 19 | 41.00 (34.00-52.00) | 45.00 (33.00-56.00) | 2.00 (-1.00-6.00) | -1.106 | 0.269 | -0.107 |
| DSST_2 | 88 | 4.50 (2.25-7.00) | 6.00 (4.00-8.00) | 1.00 (0.00-3.00) | 19 | 5.00 (4.00-6.00) | 6.00 (4.00-8.00) | 2.00 (0.00-2.00) | -0.140 | 0.889 | -0.038 |
| TMT-A | 89 | 32.20 (26.43 – 40.54) | 29.00 (24.10 – 36.94) | -4.00 (-10.43-3.25) | 19 | 39.00 (32.08 – 52.50) | 35.51 (26.50 – 50.83) | -4.84 (-14.00-8.61) | -0.391 | 0.696 | -0.038 |
| TMT-B | 87 | 70.62 (57.77 – 84.009 | 68.70 (51.00 – 80.00) | -5.09 (-15.00-6.77) | 19 | 93.43 (70.39 – 125.00) | 77.00 (55.00 – 102.00) | -17.00 (-34.20-3.98) | -1.956 | 0.050 | -0.190 |
| Subj. mental health | 96 | 5.41 (4.64 – 6.80) | 5.36 (4.39 – 6.80) | 0.00 (-0.72-0.63) | 22 | 4.09 (3.25 – 5.48) | 4.41 (3.34 – 5.11) | 0.27 (-0.79-0.84) | 0.868 | 0.386 | 0.080 |
| IQR - interquartile range, HADS-D - German Hospital Anxiety and Depression Scale, SSD-12 - Somatic Symptom Disorder - B Criteria Scale, ITQ - International Trauma Questionnaire, BFI - Brief Fatigue Inventory, FIS - Fatigue Impact Scale, MoCA - Montreal Cognitive Assessment, DSST - Digit Symbol Substitution Test (DSST_1: number of correct symbols within 90 sec., DSST_2: number of correct symbols from memory), TMT - Trail Making Test. | | | | | | | | | | | |

| **Table A18:** Groupwise comparison of psychological parameters, fatigue, and cognitive parameters of patients with pre-existing psychological disease and without a pre-existing psychological disease between timepoints T1 and T4. | | | | | | | | | | | |
| --- | --- | --- | --- | --- | --- | --- | --- | --- | --- | --- | --- |
|  | **Pre-existing psychological disease** | | | | | | | | **Between-group** | | |
|  | **No** | | | | **Yes** | | | | **difference** | | |
|  | **N** | **T1**  **Median**  **(IQR)** | **T4**  **Median**  **(IQR)** | **Δ** | **N** | **T1**  **Median**  **(IQR)** | **T4**  **Median**  **(IQR)** | **Δ** | **z** | **p** | **r** |
| HADS-D_Depression_ | 85 | 6.00 (3.00 – 9.00) | 5.00 (3.00 – 10.00) | 0.00 (-2.00-2.00) | 20 | 11.00 (8.00 – 13.00) | 10.00 (6.00 – 13.00) | -1.00 (-3.50-1.00) | -1.291 | 0.197 | -0.126 |
| HADS-D_Anxiety_ | 85 | 5.00 (3.00 – 10.00) | 5.00 (3.00 – 9.00) | -1.00 (-3.00-2.00) | 20 | 10.00 (8.00 – 14.00) | 9.50 (5.00 – 13.00) | -1.00 (-3.00-0.75) | -0.766 | 0.443 | -0,075 |
| SSD-12 | 91 | 23.00 (15.00 – 29.00) | 22.00 (12.00 – 30.00) | -2.00 (-8.00-5.00) | 21 | 29.00 (24.00 – 32.50) | 30.00 (22.00 – 35.00) | 1.00 (-5.50-7.50) | 0.873 | 0.383 | 0.083 |
| ITQ | 92 | 6.00 (2.00 – 11.00) | 3.00 (1.00 – 8.00) | -1.00 (-5.00-1.00) | 20 | 14.00 (9.00 – 18.75) | 9.50 (5.25 – 15.00) | -4.00 (-6.00-0.00) | -1.536 | 0.124 | -0.145 |
| BFI | 92 | 5.22 (4.11 – 6.39) | 6.00 (4.83 – 7.00) | 0.66 (-0.27-1.22) | 21 | 6.44 (5.33 – 7.44) | 6.00 (4.11 – 7.00) | -0.55 (-1.38-0.47) | -3.419 | <0.001 | -0.320 |
| FIS | 93 | 89.00 (71.00 – 110.00) | 96.00 (74.00 – 116.00) | 2.00 (-10.00-18.00) | 21 | 110.00 (87.00 – 118.00) | 102.00 (60.00 – 122.00) | -10.00 (-20.50-8.00) | -2.226 | 0.026 | -0.210 |
| MoCA | 91 | 27.00 (25.00-28.00) | 27.00 (26.00-28.00) | 0.00 (-1.00-2.00) | 17 | 26.00 (25.00-27.00) | 26.00 (25.00-27.00) | 0.00 (-1.50-2.00) | -0.256 | 0.798 | -0.026 |
| DSST_1 | 91 | 46.00 (37.00-53.00) | 50.00 (38.50-59.00) | 4.00 (-3.00-8.50) | 17 | 43.00 (34.00-51.50) | 47.00 (33.50-56.00) | 2.00 (-2.50-10.50) | 0.023 | 0.981 | 0.002 |
| DSST_2 | 81 | 5.00 (2.00-7.00) | 6.00 (4.00-8.00) | 1.00 (0.00-2.00) | 17 | 5.00 (3.00-6.00) | 5.00 (3.00-7.00) | 1.00 (-2.00-2.50) | -0.140 | 0.889 | -0.014 |
| TMT-A | 81 | 32.42 (26.43 – 40.54) | 28.50 (24.07 – 37.25) | -4.28 (-8.75-1.36) | 17 | 39.00 (32.54 – 52.75) | 42.23 (23.25 – 50.63) | -6.84 (-16.96-2.05) | -0.943 | 0.346 | -0.095 |
| TMT-B | 81 | 69.06 (56.50 – 83.97) | 64.00 (54.08 – 77.68) | -5.15 (19.23-6.74) | 17 | 93.43 (72.20 – 121.09) | 89.52 (56.96 – 118.25) | -9.48 (-26.44-11.25) | -0.544 | 0.586 | -0.055 |
| Subj. mental health | 92 | 5.45 (4.73 – 6.82) | 5.64 (4.55 – 6.93) | -0.04 (-0.95-1.00) | 21 | 4.55 (3.55 – 5.55) | 4.45 (3.77 – 5.32) | 0.36 (-1.00-0.95) | 0.410 | 0.682 | 0.039 |
| IQR - interquartile range, HADS-D - German Hospital Anxiety and Depression Scale, SSD-12 - Somatic Symptom Disorder - B Criteria Scale, ITQ - International Trauma Questionnaire, BFI - Brief Fatigue Inventory, FIS - Fatigue Impact Scale, MoCA - Montreal Cognitive Assessment, DSST - Digit Symbol Substitution Test (DSST_1: number of correct symbols within 90 sec., DSST_2: number of correct symbols from memory), TMT - Trail Making Test. | | | | | | | | | | | |

| **Table A19:** Groupwise comparison of psychological parameters, fatigue, and cognitive parameters of patients with pre-existing neuro-sensory disease and without a pre-existing neuro-sensory disease between timepoints T1 and T3. | | | | | | | | | | | |
| --- | --- | --- | --- | --- | --- | --- | --- | --- | --- | --- | --- |
|  | **Pre-existing neuro-sensory disease** | | | | | | | | **Between-group** | | |
|  | **No** | | | | **Yes** | | | | **difference** | | |
|  | **N** | **T1**  **Median**  **(IQR)** | **T3**  **Median**  **(IQR)** | **Δ** | **N** | **T1**  **Median**  **(IQR)** | **T3**  **Median**  **(IQR)** | **Δ** | **z** | **p** | **r** |
| HADS-D_Depression_ | 76 | 6.00 (3.25 – 10.00) | 6.00 (3.00 – 9.75) | -1.00 (-3.00-1.00) | 38 | 8.50 (5.75 – 12.00) | 8.00 (5.00 – 12.25) | 0.50 (-2.00-2.00) | 0.885 | 0.376 | 0.083 |
| HADS-D_Anxiety_ | 76 | 6.00 (3.00 – 10.75) | 6.00 (3.00 – 8.00) | -1.00 (-3.00-1.00) | 38 | 7.50 (4.00 – 11.00) | 7.00 (3.75 – 12.00) | 0.00 (-3.00-2.00) | 0.912 | 0.362 | 0.085 |
| SSD-12 | 78 | 25.00 (15.00 – 31.00) | 23.00 (15.00 – 32.25) | 0.00 (-5.00-3.00) | 40 | 25.00 (20.25 – 33.00) | 24.00 (18.25 – 34.50) | 0.00 (-7.00-6.00) | 0.196 | 0.844 | 0.018 |
| ITQ | 78 | 6.00 (2.00 – 14.00) | 5.50 (1.00 – 10.00) | -1.00 (-4.00-1.25) | 39 | 9.00 (3.00 – 14.00) | 6.00 (2.00 – 13.00) | -2.00 (-5.00-3.00) | -0.272 | 0.785 | -0.025 |
| BFI | 78 | 5.33 (4.19 – 7.00) | 5.83 (4.31 – 6.78) | 0.11 (-0.88-1.13) | 40 | 5.94 (4.92 – 6.64) | 5.89 (5.03 – 7.11) | 0.18 (-0.63-1.19) | 0.145 | 0.885 | 0.013 |
| FIS | 78 | 95.00 (71.25 – 113.25) | 94.50 (66.75 – 113.25) | -1.00 (-12.25-12.25) | 39 | 97.00 (75.00 – 114.00) | 92.00 (75.00 – 113.00) | 0.00 (-7.00-12.00) | 0.503 | 0.615 | 0.047 |
| MoCA | 72 | 27.00 (26.00-28.00) | 27.00 (26.00-28.00) | 0.00 (-2.00-1.00) | 36 | 27.00 (24.25-27.75) | 25.50 (24.00-28.00) | -1.00 (-2.00-1.00) | -1.238 | 0.216 | -0.119 |
| DSST_1 | 71 | 46.00 (37.00-53.00) | 50.00 (44.50-57.50) | 5.00 (0.00-9.00) | 36 | 45.00 (37.00-53.25) | 50.50 (43.25-56.50) | 2.00 (-2.00-10.50) | -0.555 | 0.579 | -0.054 |
| DSST_2 | 71 | 5.00 (3.00-7.00) | 7.00 (4.00-8.00) | 2.00 (0.00-3.00) | 36 | 5.00 (2.25-6.00) | 5.00 (3.00-7.75) | 0.50 (-1.00-3.00) | -1.516 | 0.130 | -0.147 |
| TMT-A | 72 | 32.59 (26.28 – 41.00) | 30.70 (24.35 – 36.97) | -4.54 (-11.86-4.07) | 36 | 36.90 (28.03 – 49.50) | 30.75 (24.65 – 42.53) | -2.58 (-10.75-3.00) | 0.495 | 0.620 | 0.048 |
| TMT-B | 72 | 72.55 (60.47 – 88.75) | 67.00 (49.00 – 79.75) | -7.86 (-17.55-4.38) | 34 | 70.81 (55.95 – 92.66) | 73.00 (59.70 – 90.75) | -1.74 (-12.00-20.75) | 1.506 | 0.132 | 0.146 |
| Subj. mental health | 78 | 5.27 (4.16 – 6.59) | 5.09 (4.36 – 6.75) | 0.09 (-0.63-0.65) | 40 | 5.32 (4.45 – 6.20) | 5.00 (3.66 – 6.36) | -0.22 (-0.97-0.70) | -1.129 | 0.259 | -0.104 |
| IQR - interquartile range, HADS-D - German Hospital Anxiety and Depression Scale, SSD-12 - Somatic Symptom Disorder - B Criteria Scale, ITQ - International Trauma Questionnaire, BFI - Brief Fatigue Inventory, FIS - Fatigue Impact Scale, MoCA - Montreal Cognitive Assessment, DSST - Digit Symbol Substitution Test (DSST_1: number of correct symbols within 90 sec., DSST_2: number of correct symbols from memory), TMT - Trail Making Test. | | | | | | | | | | | |

| **Table A20:** Groupwise comparison of psychological parameters, fatigue, and cognitive parameters of patients with pre-existing neuro-sensory disease and without a pre-existing neuro-sensory disease between timepoints T1 and T4. | | | | | | | | | | | |
| --- | --- | --- | --- | --- | --- | --- | --- | --- | --- | --- | --- |
|  | **Pre-existing neuro-sensory disease** | | | | | | | | **Between-group** | | |
|  | **No** | | | | **Yes** | | | | **difference** | | |
|  | **N** | **T1**  **Median**  **(IQR)** | **T4**  **Median**  **(IQR)** | **Δ** | **N** | **T1**  **Median**  **(IQR)** | **T4**  **Median**  **(IQR)** | **Δ** | **z** | **p** | **r** |
| HADS-D_Depression_ | 72 | 6.00 (3.25 – 10.00) | 5.00 (3.00 – 10.00) | -1.00 (-3.00-2.00) | 33 | 8.00 (5.00 – 11.50) | 7.00 (5.00 – 13.00) | 1.00 (-1.50-3.00) | 1.536 | 0.125 | 0.150 |
| HADS-D_Anxiety_ | 72 | 6.00 (3.25 – 11.00) | 5.00 (3.00 – 10.00) | -1.00 (-3.75-1.75) | 33 | 7.00 (4.00 – 10.50) | 6.00 (5.00 – 9.50) | 0.00 (-2.00-1.50) | 1.643 | 0.100 | 0.160 |
| SSD-12 | 75 | 24.00 (15.00 – 29.00) | 23.00 (16.00 – 30.00) | -2.00 (-7.00-5.00) | 37 | 25.00 (20.50 – 33.50) | 24.00 (18.50 – 34.00) | -1.00 (-10.00-7.00) | 0.000 | 1.000 | 0.000 |
| ITQ | 75 | 6.00 (2.00 – 13.00) | 3.00 (1.00 – 9.00) | -2.00 (-6.00-1.00) | 37 | 9.00 (3.50 – 14.00) | 5.00 (1.50 – 12.00) | -2.00 (-5.00-0.00) | -0.037 | 0.970 | -0.004 |
| BFI | 76 | 5.33 (4.83 – 6.67) | 5.89 (4.47 – 6.75) | 0.44 (-0.41-1.11) | 38 | 5.89 (4.83 – 6.56) | 6.22 (5.00 – 7.11) | 0.33 (-0.80-1.22) | -0.141 | 0.888 | -0.013 |
| FIS | 76 | 94.50 (69.75 – 111.00) | 98.00 (69.00 – 117.00) | 0.00 (-11.00-13.00) | 36 | 97.00 (75.75 – 115.50) | 96.00 (79.75 – 118.75) | 3.00 (-14.75-17.75) | 0.336 | 0.737 | 0.032 |
| MoCA | 76 | 27.00 (25.25-28.00) | 27.00 (26.00-29.00) | 0.00 (-1.00-2.00) | 30 | 27.00 (24.75-27.25) | 27.00 (24.00-28.00) | 0.00 (-2.00-1.00) | -0.901 | 0.368 | -0.091 |
| DSST_1 | 68 | 46.00 (37.00-53.75) | 49.50 (40.25-60.00) | 3.50 (-2.75-10.50) | 30 | 46.00 (35.75-53.00) | 49.00 (36.00-56.00) | 2.00 (-3.25-7.00) | -0.853 | 0.394 | -0.086 |
| DSST_2 | 6 | 5.00 (3.00-7.00) | 6.00 (4.00-8.00) | 1.00 (0.00-2.00) | 30 | 5.00 (2.00-6.00) | 6.00 (4.00-8.00) | 1.50 (-0.25-3.25) | 0.594 | 0.553 | 0,060 |
| TMT-A | 68 | 32.88 (26.28 – 41.00) | 28.37 (23.42 – 36.21) | -5.56 (-9.00-0.99) | 30 | 37.93 (28.09 – 50.50) | 32.75 (24.49 – 51.40) | -3.55 (-13.04-6.52) | 0.597 | 0.550 | 0.060 |
| TMT-B | 68 | 71.11 (59.84 – 84.75) | 64.00 (54.08 – 77.23) | -7.41 (-21.91-4.82) | 30 | 69.00 (55.59 – 99.25) | 73.24 (56.00 – 104.50) | 3.61 (-13.22-25.50) | 2.297 | 0.022 | 0.232 |
| Subj. mental health | 76 | 5.36 (4.55 – 6.73) | 5.27 (4.48 – 6.64) | 0.04 (-0.95-1.00) | 37 | 5.36 (4.50 – 6.27) | 5.18 (4.09 – 6.27) | 0.00 (-1.02-0.95) | -0.236 | 0.814 | -0.022 |
| IQR - interquartile range, HADS-D - German Hospital Anxiety and Depression Scale, SSD-12 - Somatic Symptom Disorder - B Criteria Scale, ITQ - International Trauma Questionnaire, BFI - Brief Fatigue Inventory, FIS - Fatigue Impact Scale, MoCA - Montreal Cognitive Assessment, DSST - Digit Symbol Substitution Test (DSST_1: number of correct symbols within 90 sec., DSST_2: number of correct symbols from memory), TMT - Trail Making Test. | | | | | | | | | | | |

| **Table A21:** Groupwise comparison of psychological parameters, fatigue, and cognitive parameters of patients with pre-existing musculoskeletal disease and without a pre-existing musculoskeletal disease between timepoints T1 and T3. | | | | | | | | | | | |
| --- | --- | --- | --- | --- | --- | --- | --- | --- | --- | --- | --- |
|  | **Pre-existing musculoskeletal disease** | | | | | | | | **Between-group** | | |
|  | **No** | | | | **Yes** | | | | **difference** | | |
|  | **N** | **T1**  **Median**  **(IQR)** | **T3**  **Median**  **(IQR)** | **Δ** | **N** | **T1**  **Median**  **(IQR)** | **T3**  **Median**  **(IQR)** | **Δ** | **z** | **p** | **r** |
| HADS-D_Depression_ | 41 | 7.00 (3.50 – 10.50) | 5.00 (3.00 – 9.50) | -1.00 (-4.00-1.00) | 73 | 8.00 (4.00 – 11.00) | 7.00 (3.00 – 11.00) | 0.00 (-2.00-2.00) | 1.305 | 0.192 | 0.122 |
| HADS-D_Anxiety_ | 41 | 6.00 (3.00 – 10.50) | 4.00 (2.50 – 7.00) | -1.00 (-3.00-1.00) | 73 | 7.00 (4.00 – 11.00) | 7.00 (3.50 – 10.00) | 0.00 (-3.00-2.00) | 0.433 | 0.665 | 0.041 |
| SSD-12 | 41 | 24.00 (16.50 – 27.00) | 20.00 (14.50 – 29.50) | 0.00 (-8.00-5.00) | 77 | 25.00 (17.00 – 34.00) | 25.00 (18.00 – 34.50) | 0.00 (-6.00-3.00) | 0.023 | 0.982 | 0.002 |
| ITQ | 41 | 7.00 (3.00 – 13.50) | 5.00 (1.00 – 10.00) | -1.00 (-4.00-1.00) | 76 | 6.50 (2.25 – 14.00) | 6.00 (1.25 – 11.00) | -1.00 (-4.00-2.00) | 0.722 | 0.471 | 0.067 |
| BFI | 41 | 5.22 (4.17 – 6.61) | 5.78 (4.83 – 6.61) | 0.15 (-0.77-1.16) | 77 | 5.89 (4.78 – 6.83) | 5.89 (4.89 – 7.11) | 0.11 (-0.77-1.16) | -0.328 | 0.743 | -0,030 |
| FIS | 41 | 97.00 (76.50 – 115.00) | 94.00 (77.00 – 113.00) | 0.00 (-11.50-14.50) | 76 | 95.50 (72.00 – 111.75) | 93.00 (66.25 – 113.00) | 0.00 (-10.75-11.75) | -0.209 | 0.835 | -0.019 |
| MoCA | 38 | 27.00 (25.00-28.25) | 26.00 (25.00-28.00) | 0.00 (-2.25-1.00) | 70 | 27.00 (25.00-28.00) | 27.00 (24.75-28.00) | 0.00 (-2.00-1.00) | 0.156 | 0.876 | 0.015 |
| DSST_1 | 37 | 46.00 (37.00-53.00) | 50.00 (44.50-57.50) | 3.00 (-1.00-9.00) | 70 | 45.00 (37.00-53.25) | 50.50 (43.25-56.50) | 4.00 (-1.00-8.25) | -0.089 | 0.929 | -0.009 |
| DSST_2 | 37 | 5.00 (3.00-7.00) | 7.00 (4.00-8.00) | 1.00 (-0.50-2.00) | 70 | 5.00 (2.00-6.00) | 6.00 (4.00-8.00) | 1.50 (0.00-3.00) | 1.073 | 0.283 | 0.103 |
| TMT-A | 38 | 29.73 (25.03 – 39.04) | 30.60 (22.60 – 37.04) | -2.34 (-11.18-6.12) | 70 | 35.06 (29.14 – 45.86) | 30.85 (24.75 – 38.93) | -4.37 (-11.38-2.85) | -0.968 | 0.333 | -0.093 |
| TMT-B | 37 | 71.15 (58.96 – 90.50) | 69.20 (51.90 – 84.25) | -8.04 (-18.22-6.88) | 69 | 72.00 (59.50 – 88.94) | 69.90 (52.11 – 82.70) | -5.09 (-17.00-4.95) | 0.265 | 0.791 | 0.026 |
| Subj. mental health | 41 | 5.45 (4.55 – 6.77) | 5.00 (4.27 – 6.64) | -0.18 (-1.00-0.45) | 77 | 5.18 (4.14 – 6.27) | 5.09 (4.32 – 6.64) | 0.18 (-0.72-0.81) | 1.685 | 0.092 | 0.155 |
| IQR - interquartile range, HADS-D - German Hospital Anxiety and Depression Scale, SSD-12 - Somatic Symptom Disorder - B Criteria Scale, ITQ - International Trauma Questionnaire, BFI - Brief Fatigue Inventory, FIS - Fatigue Impact Scale, MoCA - Montreal Cognitive Assessment, DSST - Digit Symbol Substitution Test (DSST_1: number of correct symbols within 90 sec., DSST_2: number of correct symbols from memory), TMT - Trail Making Test. | | | | | | | | | | | |

| **Table A22:** Groupwise comparison of psychological parameters, fatigue, and cognitive parameters of patients with pre-existing musculoskeletal disease and without a pre-existing musculoskeletal disease between timepoints T1 and T4. | | | | | | | | | | | |
| --- | --- | --- | --- | --- | --- | --- | --- | --- | --- | --- | --- |
|  | **Pre-existing musculoskeletal disease** | | | | | | | | **Between-group** | | |
|  | **No** | | | | **Yes** | | | | **difference** | | |
|  | **N** | **T1**  **Median**  **(IQR)** | **T4**  **Median**  **(IQR)** | **Δ** | **N** | **T1**  **Median**  **(IQR)** | **T4**  **Median**  **(IQR)** | **Δ** | **z** | **p** | **r** |
| HADS-D_Depression_ | 38 | 6.50 (3.00 – 10.00) | 5.00 (3.00 – 10.00) | -1.00 (-3.00-2.00) | 67 | 7.00 (4.00 – 11.00) | 7.00 (4.00 – 11.00) | 0.00 (-2.00-2.00) | 1.189 | 0.235 | 0.116 |
| HADS-D_Anxiety_ | 38 | 6.00 (3.00 – 10.25) | 4.00 (2.75 – 8.00) | -1.00 (-2.25-1.00) | 67 | 7.00 (4.00 – 11.00) | 6.00 (4.00 – 10.00) | 0.00 (-3.00-2.00) | 0.579 | 0.562 | 0.057 |
| SSD-12 | 38 | 24.00 (15.00 – 26.00) | 20.00 (10.50 – 28.25) | -1.00 (-2.25-1.00) | 74 | 25.00 (17.00 – 33.00) | 24.50 (18.00 – 32.00) | -2.50 (-7.00-5.25) | 0.271 | 0.787 | 0.026 |
| ITQ | 39 | 7.00 (4.00 – 14.00) | 4.00 (1.00 – 9.00) | -2.00 (-6.00-1.00) | 73 | 6.00 (2.00 – 12.50) | 4.00 (1.00 – 9.00) | -1.00 (-5.00-0.009 | 0.892 | 0.372 | 0.084 |
| BFI | 40 | 5.22 (4.14 – 6.64) | 5.83 (4.25 – 6.64) | 0.33 (-0.75-1.19) | 74 | 5.61 (4.67 – 6.58) | 6.11 (5.00 – 7.03) | 0.55 (-0.58-1.22) | 0.297 | 0.766 | 0.028 |
| FIS | 40 | 96.00 (75.75 – 114.00) | 96.00 (70.00 – 118.00) | -2.00 (-12.00-17.00) | 72 | 95.50 (72.00 – 111.00) | 98.50 (74.00 – 116.75) | 1.00 (-12.75-14.00) | 0.395 | 0.693 | 0.037 |
| MoCA | 36 | 27.00 (25.25-28.00) | 27.00 (26.00-28.009 | 0.00 (-1.00-1.00) | 62 | 27.00 (25.00-28.00) | 27.00 (26.00-29.00) | 0.00 (-1.00-2.00) | 0.242 | 0.809 | 0.024 |
| DSST_1 | 36 | 46.00 (36.25-53.00) | 48.50 (38.50-64.25) | 2.50 8-3.75-11.00) | 62 | 46.00 (37.00-53.00) | 49.50 (37.00-58.00) | 4.00 (-2.00-8.00) | 0.214 | 0.831 | 0.022 |
| DSST_2 | 36 | 5.00 (3.00-7.00) | 5.50 (4.00-7.75) | 1.00 (-1.00-2.00) | 62 | 5.00 (2.00-6.00) | 6.00 (4.00-8.00) | 1.50 (0.00-3.00) | 1.505 | 0.132 | 0.152 |
| TMT-A | 36 | 29.73 (24.56 – 39.11) | 29.33 (22.31 – 44.31) | -4.07 (-8.04-1.14) | 62 | 35.46 (29.14 – 45.86) | 28.90 (24.16 – 39.50) | -5.68 (-11.44-2.02) | -1.091 | 0.275 | -0.110 |
| TMT-B | 36 | 71.11 (57.33 – 91.25) | 66.75 (55.26 – 84.98) | -7.03 (-15.34-4.67) | 62 | 70.70 (57.94 – 85.75) | 65.27 (54.24 – 87.00) | -2.50 (-21.96-8.20) | 0.475 | 0.635 | 0.048 |
| Subj. mental health | 39 | 5.45 (4.55 – 6.73) | 5.27 (4.55 – 7.09) | 0.18 (-0.59-0.55) | 74 | 5.32 (4.45 – 6.41) | 5.23 (4.34 – 6.43) | 0.00 (-1.00-1.00) | -0.335 | 0.737 | -0.032 |
| IQR - interquartile range, HADS-D - German Hospital Anxiety and Depression Scale, SSD-12 - Somatic Symptom Disorder - B Criteria Scale, ITQ - International Trauma Questionnaire, BFI - Brief Fatigue Inventory, FIS - Fatigue Impact Scale, MoCA - Montreal Cognitive Assessment, DSST - Digit Symbol Substitution Test (DSST_1: number of correct symbols within 90 sec., DSST_2: number of correct symbols from memory), TMT - Trail Making Test. | | | | | | | | | | | |
